# Self-health promotion: A study on the mode of acquiring sports health knowledge and skills among older adults members of sports communities

**DOI:** 10.1371/journal.pone.0304814

**Published:** 2024-07-11

**Authors:** Ye Wei

**Affiliations:** College of physical education, Henan University of Science and Technology, Luoyang, China; University of Tartu, ESTONIA

## Abstract

**Objective:**

The research aims to construct a mode and the pathway relationships of acquiring sports health knowledge and skills among members of older adults sports communities.

**Method:**

The research was primarily conducted through random sampling, purposive sampling, and questionnaire surveys. A sample of 457 older adults from Luoyang City was selected as the research subjects. Exploratory analysis and Structural Equation Modeling (SEM) were conducted by employing SPSS 26.0 and Amos 26.0 software(Exploratory analysis and structural equation analysis).

**Results:**

The study indicates that the influence of older adults sports community culture on the pursuit of sports health knowledge and skills is statistically significant (*β* = 0.69, *P*<0.001); the influence of sports community culture on the motivation to enhance sports health knowledge and skills is statistically significant (*β* = 0.32, *P*<0.001); the influence of the pursuit of sports health knowledge and skills on the motivation to enhance these knowledge and skills is statistically significant (*β* = 0.47, *P*<0.001); the influence of the motivation to enhance sports health knowledge and skills on the behavior of acquiring these knowledge and skills is statistically significant (*β* = 0.60, *P*<0.001); both the pursuit of sports health knowledge and skills and the motivation to enhance these knowledge and skills serve as mediating variables.

**Conclusion:**

The more harmonious and positive the sports community culture is, the stronger the sense of pursuing health and the motivation to acquire knowledge are among older adults. The stronger the health pursuit among older adults, the higher their motivation to enhance sports health knowledge and skills shows. Furthermore, the higher the motivation of older adults to enhance sports health knowledge and skills is, the more efficient their behavior in acquiring these knowledge and skills becomes. The motivation to enhance sports health knowledge and skills serves as a complete mediating variable in this process.

## 1. Introduction

### 1.1 Research background

The physical health of older adults remains a hot topic in academic research. Enhancing the physical health of older adults is not only a priority for the nation but also a long-term issue relevant to the future development of the country and its people. Regarding health promotion, it is widely recognized that "sports" are essential to promoting health, and their role and benefits in improving the physical health of older adults have been widely recognized. In real life, many older adults choose group-based exercises. The topic of older adult health is a core part of sports community discussions. The storage and application of sports health knowledge and skills are primary means to promote older adults’ health, and researchers on this subject argue that further research is necessary on this subject. Therefore, they proposed to explore the mode for acquiring sports health knowledge and skills by members of sports communities. The research includes four parts: community culture, the pursuit of sports health knowledge and skills, motivations for enhancing sports health knowledge and skills, and behavior in acquiring sports health knowledge and skills. Upon reviewing existing literature, the researchers found that few articles once used Structural Equation Modeling (SEM) to explore the relationships among these four parts. Consequently, the study started from this gap and proposed to further elaborate the self-health promotion process of older adults by applying the SEM. By analyzing the experience of members of sports communities, the research aims to explore the pattern of acquiring sports health knowledge and skills among older adults in their self-health promotion process, and discuss the formation mode of self-health promotion among older adults from this perspective.

### 1.2 Literature review

#### 1.2.1 The influence of sports community culture on the pursuit of sports health knowledge and skills

Community culture is the soul of the sustainable development of communities, a unique and dynamic cultural phenomenon. It not only satisfies the needs of individual belonging and identity but also drives the development of communities. In modern society, with the proliferation of the Internet and the rise of social media, the influence of community culture is gradually expanding and has become one significant approach to the prosperity of social culture, giving rise to various online communities. Rehabilitation-focused online communities have become an essential daily communication channel for patients with chronic diseases. A large number of non-healthy individuals gather here to spread health information, share experiences in rehabilitation and treatment, and offer mutual comfort and encouragement. On the one hand, this trend has somewhat weakened the authority and discourse power of medical institutions; on the other hand, it helps alleviate patients’ anxiety and fear in real life. All these have contributed to transforming online communities into a "psychological massage room" for fellow patients. Online communities are characterized by shared rehabilitation goals and self-health promotion intentions [1]. Sports communities are interpersonal network platforms with the pursuit of health as their goal and functional exercise as their method. Sports not only uplift individuals’ moods but also serve as a medium for communication among community members, steering and regulating individual behaviors toward achieving physical and mental health and fostering a sense of unity. Additionally, the compulsory community norms also contribute to motivating older adults to persist in improving their exercise skills [2]. The sports community culture of older adults emphasizes values of health, happiness, and friendship. Such a cultural atmosphere can inspire older adults to pursue sports health knowledge and skills [3]. In sports communities, older adults learn about the importance of sports for physical and mental health through mutual exchange and sharing. This recognition encourages them to take part in various sports actively, improving their sports skills and health literacy [4]. The development of communities requires maintenance by their members. The maintenance of sports communities requires a comprehensive consideration of community orientation, content, event organization, rules, and member needs. Only through constant efforts and innovation can a vibrant, harmonious, and friendly sports community be built. All these joint efforts can promote the achievement of the community’s collective and individual goals [5]. Some researchers hold that to keep the aging population vibrant, it is necessary to ensure that older adults can lead a healthy and long life. This can be achieved by utilizing artificial intelligence (AI) and new internet technologies to track older adults’ physical activities, promote their social engagement, fulfill their full potential, and seek out the significance of life [6]. Based on the comprehensive analysis of the literature, it can be observed that sports community culture provides members with a platform for learning and communication. On this platform, participants can acquire various sports health knowledge and skills, and learn about different exercise methods and skills. By engaging in community sports, they can also make like-minded friends, mutually encourage and support each other, achieve self-fulfillment goals, and enhance health literacy.

#### 1.2.2 The influence of sports community culture on the motivation to enhance sports health knowledge and skills

An individual’s motivation is instrumental in determining behavior. It not only serves as a driving force but also determines the direction and persistence of behavior. The primary goal of older adults in acquiring health knowledge is to enhance their health because of their instinctive desire for health and longevity. With the advancement of media communication, older adults’ health awareness has also increased accordingly, reinforcing their health beliefs [7]. Some researchers have discussed the mechanism that influences health behaviors from five perspectives: individual, interpersonal, organization, community, and policy. They argue that the persistence of health behaviors is based on the improvement of health awareness and the updating of scientific sports health knowledge [8]. Xin [9] pointed out that urban older adults were motivated to gain a voice in health literacy, which in turn drove them to acquire sports health knowledge actively. Consequently, they engaged in physical exercise with a well-planned approach to acquiring sports health knowledge and skills. The influence of sports community culture on motivation is multidimensional; it can inspire and shape individual motivation from various perspectives. The contagiousness of sports community culture can profoundly impact participants’ motivations. As a group-oriented culture, sports community culture encompasses a wealth of values, beliefs, spirit, moral habits, and norms. Some researchers, taking football fans as an example, suggested that the chain of interactive rituals among fans facilitated the formation of new motivations and influenced their future actions, which would exert a profound influence on their thoughts and behaviors [10]. Furthermore, some researchers found that members of the Yangge dance (“Yangge dance”, a popular rural folk dance in China) community could also be motivated through social comparison and competition. These dancers often showcased their strengths and skills through comparison and competition, a process that could enhance their competitive spirit and desire to excel, thereby enhancing their motivation [11].

#### 1.2.3 The pursuit of sports health knowledge and skills has a statistically significant impact on the motivation to enhance sports health knowledge and skills

When individuals have a strong desire for sports health knowledge and skills, this pursuit can often be transformed into motivation, which in turn drives them to actively acquire and improve relevant knowledge and skills [12]. Luan Jun et al. held that the motivation of older adults to engage in Taijiquan stemmed from their pursuit of health as well as their interest in the skills involved in practicing Taijiquan [13]. The pursuit of health knowledge among older adults can make them aware of their gaps in this field, thereby generating a desire for learning [14]. Moreover, by comparing with others, older adults may also realize their gaps in sports health knowledge and skills, which in turn creates a motivation to learn [15]. Some researchers believe that self-presentation is correlated with motivation for sports and health. By engaging in sports, older adults demonstrate their positive attitude towards life and their emphasis on health, which not only benefits enhance their sense of self-worth, deepens their influence on others, and broadens their social circle, but also fosters the pursuit of higher quality of life and greater spiritual fulfillment [16]. Xiong Haiqin posited that sports community norms and the cultural climate of sports could significantly motivate older adults to engage in physical exercise, enhance the sense of self-efficacy among community members, clearly define their exercise goals, and further stimulate their motivation to engage in exercise [17]. Additionally, the sharing of personal health data by members in online communities forms a basis for social comparison, which is beneficial not only for enhancing the motivation to pursue health among members but also for improving individual health literacy [18].

#### 1.2.4 The influence of the motivation to enhance sports health knowledge and skills on the behavior of acquiring sports health knowledge and skills

The motivation to enhance sports health knowledge and skills arises from an individual’s concern and pursuit of health and physical activities. When individuals recognize the importance of sports health knowledge and skills, they develop a strong motivation to acquire these knowledge and skills, gradually forming a habit [19]. Buchmann and his team pointed out that critical health literacy involves the critical evaluation of health information, and the conscious action to create an environment that promotes one’s own and others’ health. This allows individuals in a community to change their lifestyles and implement effective health behaviors [20]. As community members browse digital health information on the internet, the recognition of disparities with others can prompt an epiphany regarding health knowledge, which in turn leads them to reevaluate their behaviors. When recognizing the gap in knowledge deficiencies widens, members may begin to strive to acquire health knowledge, thereby improving their health literacy [21]. Motivation and behavior are not necessarily linearly correlated. Zhang Lu posited that there was a specific relationship between "knowledge, attitude, and practice" among older adults intellectuals in universities [22]. They are highly willing to acquire health knowledge and access a wealth of health information. However, there is a noticeable gap in their health behaviors, with some older adults intellectuals showing weaker abilities in executing health actions. This indicates that older adults intellectuals in universities are not efficiently applying health knowledge in their daily lives, and their ability to transform knowledge into health behaviors is unsatisfactory. Older adults acquire information mainly through WeChat’s "Moment," and secondly through "group chat." They tend to be more inclined to open WeChat articles with suggestive or authoritative titles, while few will open articles with sentimental titles [23], indicating that the behavior of older adults in acquiring knowledge exhibits a cognitive tendency.

According to Cognitive Development Theory, the study of older adults’ acquisition of sports health knowledge and skills in the process of self-health promotion can be viewed as the development of their cognitive schemas. In this process, individuals utilize sports health knowledge and skills to construct their physical health. This process will dynamically change with the development of older adults’ cognitive schemas. It also indicates the dynamic shifts between older adults’ health motivation and health behaviors.

#### 1.2.5 The pursuit of health knowledge and skills, as well as the motivation to enhance physical health knowledge and skills serves as mediating variables

To construct a Structural Equation Modeling (SEM) for the acquisition of sports health knowledge and skills among sports community members, it is essential to establish the path relationships based on relevant theory and empirical research findings. Upon reviewing previous research findings, scholars have explored health promotion from the perspectives of individuals’ intrinsic factors and external environmental elements. In terms of theory, based on the Behavioral Learning Theory Model "External Stimuli → Needs → Motivation → Behavior," it can be inferred that an individual’s learning behaviors are stimulated by numerous factors in conjunction with their own needs, and motivated by driving force. Learning motivation is externally driven, with needs being the cause and external stimuli being the condition; motivation is the interplay between cause and condition, and behavior acts as the outcome. The Behavioral Learning Theory considers an individual’s needs and motivations as mediating variables. Empirically, the habit of older adults actively acquiring sports health knowledge and skills is influenced by various factors. Yao [24], in her research on older adults communities, found that the culture of the older adults team and personal pursuits were prerequisites for actively acquiring health knowledge and skills. For participants classified as "disabled, the team culture influences their mindset towards health promotion from the way of exercising to the pursuit of the infinite, from the motivation for pursuing physical health to the formation of healthy behavioral habits. All stems from participants’ motivation to acquire health knowledge and skills. And this motivation will be subtly transformed into self-professional growth or health promotion behaviors. Numerous scholars have also demonstrated the mediating mechanism of sports motivation between sports culture and behaviors [25, 26], as well as the linear model relationship of "Environment—Basic Psychological Needs—Behavioral Motivation—Behavioral Engagement" from cultural performance to collective action in communities [27, 28].

### 1.3 Research hypotheses

Based on the literature analysis, this section posits hypotheses concerning the research objectives of older adults sports communities.

H1. The sports community culture has a statistically significant influence on the pursuit of sports health knowledge and skills.
H2. The sports community culture has a statistically significant influence on the motivation to enhance sports health knowledge and skills.
H3. The pursuit of sports health knowledge and skills has a statistically significant influence on the motivation to enhance sports health knowledge and skills.
H4. The motivation to enhance sports health knowledge and skills has a significant influence on behaviors of acquiring sports health knowledge and skills.
H5. The pursuit of sports health knowledge and skills, and the motivation to enhance sports health knowledge and skills act as mediating variables in the pathway relationships, with statistical significance.

## 2. Measures

### 2.1 Research procedure and demographic characteristics

The researcher discussed the research framework with four experts. The researcher independently designed the research plan for the project based on the results of the discussion. Regarding the age classification of older adults, the World Health Organization (WHO) defines young older adults as those aged 60~74 years. However, the current global standard for categorizing older adults varies. In developing countries, people aged 60 and over are considered as older adults, while in the developed countries like Europe and America, people aged 65 and over are considered as older adults. The Geriatrics Division of the Chinese Medical Association stipulates that people aged 60 and over are considered as older adults.

Standardized training was given to research team members to ensure their understanding of the significance and methodology of this survey. And they were also equipped with basic communication skills(Only explain the content of the questionnaire, assist older adults with difficulties in filling out questionnaires), and randomly interview older adults according to the interview outline after completing the questionnaires. Before conducting the survey, the purpose of the research was explained to older adults respondents to seek their consent. During the deployment phase, on-site questionnaires were distributed. Older adults respondents who were capable of independently filling out the questionnaire were allowed to finish by themselves, while assistance was provided by research team members for those who could not complete it by themselves. Throughout the survey, questions were posed appropriately and any suggestive communication with older adults respondents was prohibited, thereby ensuring the authenticity and integrity of the survey results. Upon completion, questionnaires were meticulously numbered and recorded by researchers and then reviewed by quality controllers.

In Luoyang City, twelve communities were randomly selected for the study. Upon securing permission from the local community residents’ committees, data on the older adults sports communities were collected. From September to November of 2023, random sampling was employed to survey the older adults among these twelve communities, with a total of 650 older adults sampled. Subsequently, a purposive sampling method was employed to remove those who did not meet the criteria, leaving a final sample of 505 eligible older adults. A written informed consent letter was obtained from these older adults respondents, and 36 members from 12 sports communities were selected as interviewees by applying the snowball sampling method.

Inclusion criteria: (1) Older adults residing in Luoyang city for at least 1 year; (2) Age: ≥60 years; (3) Well-informed about and consenting to the research topic, adhering to the principle of voluntary participation; (4) Proficient in communication, free of mental disorders, and possessing a reasonable level of physical activity ability; (5) Older adults respondents must have at least 6 months’ experience in community sports activities.

Exclusion criteria: (1) Severe hearing or vision impairment; (2) Cognitive impairments (communication difficulties) or lack of mobility.

Contents of the older adults background material survey: Gender (Female, male. Assigned values in the order 1, 2), age (age groups: 60~62, 63~64, 65~66, ≥67. Assigned values in the order 1, 2, 3, 4), educational background (education level categories: Primary school education or below, Middle (or high) school education, Junior college education or above. Assigned values in the order 1, 2 and 3 respectively), health status (health status categories: unhealthy status: feeling unwell or ill in the last eight weeks; healthy status. Assigned values in the order 1, 2). Number of years devoted to sports(Number of years: 0.6, 1, 2, ≥3. Assigned values in the order 1, 2, 3, 4).

The survey was conducted applying a time-limited response and was subsequently retrieved, with first-hand data collected through interviews and active participation. A total of 505 questionnaires were distributed, yielding 476 responses. After filtering out incomplete questionnaires, 457 valid responses were obtained, consisting of 291 from male respondents and 166 from female respondents.

### 2.2 Research tools

Based on the research findings of Wei [29], a revised survey scale has been developed by referring to the opinions of eight senior sports community members and four sports experts. The *"Sports Community Culture Scale"* encompasses four dimensions: community norms, social capital, community attraction, and community maintenance, each consisting of four item factors. The Cronbach’s alpha value is 0.885, indicating the high reliability of this scale.

The *"Motivation to Enhance Sports Health Knowledge and Skills Scale*" encompasses two dimensions: the motivation to acquire novel sports health knowledge and skills, as well as the perceived gap in sports health knowledge and skills, each consisting of six item factors. The Cronbach’s alpha value is 0.908, indicating the high reliability of this scale.

The *"Behavior of Acquiring Sports Health Knowledge and Skills Scale"* encompasses four dimensions: behavioral involvement, cognitive involvement, emotional involvement, and socio-psychological involvement. The behavioral involvement dimension includes five item factors, while the other dimensions each consist of four item factors. The Cronbach’s alpha value is 0.900, indicating the high reliability of this scale.

The *"Health Knowledge and Skill Pursuit Scale"* encompasses three dimensions: self-efficacy, self-enrichment, and self-expression, each consisting of four item factors. The Cronbach’s alpha value is 0.864, indicating the high reliability of this scale.

The above scales employ the Likert 1–5 scoring system, with scores ranging from 1 (strongly disagree) to 5 (strongly agree). A higher score reflects a higher degree of agreement among individuals.

### 2.3 Statistical analysis

Data was processed by SPSS 26.0 and Amos 26.0 software (Exploratory analysis and structural equation analysis) and the significance level for all variables was set at α = 0.05.

### 2.4 Ethics approval and consent to participate

The research project does not include any animal experiments or human drug experiments. It is a public welfare project, and the informed consent is obtained before the research. After discussion by the ethics committee of College of physical education, Henan University of science and technology, the project research was approved (Project No: 20230912). Before participating in the study, each participant’s written informed consent was solicited.

## 3. Results

### 3.1 Demographic characteristics of older adults

The demographic characteristics of older adults (as shown in Table 1) are as follows: The majority of participants in the activities of sports communities are female (accounting for 63.7%), with a relatively balanced distribution across age groups. A higher percentage of participants have achieved a junior college degree or above (accounting for 51.8%). The majority of participants (accounting for 57.7%) have been engaged in sports activities for 1–2 years.

**Table 1:** Demographic characteristics of older adults.

Population situation	Factor	Number of individuals (%)	Population situation	Factor	Number of individuals (%)
Gender	Female	291(63.7%)	Age (in years)	60–62	127(27.8%)
	Male	166(36.3%)		63–64	113(24.7%)
Health condition	Health	238(52.1%)		65–66	117(25.6%)
	Nonhealthy	219(47.9%)		≥67	100(21.9%)
Educational background	Primary school education or below	63(13.8%)	Number of years devoted to sports	0.6	79(17.3%)
	Middle (or high) school education	157(34.4%)		1	130(28.4%)
	Junior college education or above	237(51.8%)		2	134(29.3%)
				≥3	114(24.9%)

### 3.2 The exploratory analysis of the survey data

Table 2 Convergent validity test and principal factor analysis of the scales were conducted by employing SPSS 26.0 software. The accumulated interpretation variance of the four factors extracted from the scales is 50% higher than the standard value, indicating good explanatory validity. When the absolute value of the skewness coefficient exceeds 2 and the absolute value of the kurtosis coefficient exceeds 5, it is considered abnormal. In this study, skewness and kurtosis comply with statistical standards, the data presents a normal distribution. Through principal factor analysis, all item factor loadings are 50% higher than the standard value, indicating good convergent validity among all scales. The reliability of composite factors is 60% higher than the standard value, and the average variance extraction is 50% higher than the standard value, indicating good convergent validity.

**Table 2:** The exploratory analysis of the survey data.

Scale	Accumulated Interpretation Variance	Skewness	Kurtosis	Factor loading	Average variance extraction	Combinatorial reliability
Sports community culture	61.758%	0.027–0.604	0.084–1.653	0.586–0.802	0.5≈0.51	0.80
Motivation to Enhance Sports and Health Knowledge and Skills	64.499%	0.382–0.767	0.011–0.741	0.563–0.869	0.5≈0.52	0.69
Pursuit of sports health knowledge and skills	58.279%	0.009–0.331	0.005–0.531	0.576–0.829	0.5≈0.49	0.74
Behavior of acquiring sports health knowledge and skills	61.467%	0.013–1.380	0.129–1.166	0.569–0.840	0.5≈0.48	0.79

Note: According to statistical rules, the values of Average Variance Extraction (AVE) and Combinatorial Reliability (CR) are one of the methods for evaluating SEM models, but not the only criterion. In practical applications, other indicators should be considered comprehensively. The values of AVE and CR are related to the degree of understanding of the survey subjects. Wu Minglong (2009) believes that the ideal value of AVE should be greater than 0.5, with a threshold of 0.36–0.5 being acceptable, and the perfect value of CR should be greater than 0.6.

### 3.3 The analysis of factor fit in the Structural Equation Modeling (SEM)

Table 3 Utilizing the maximum likelihood method to estimate the non-standardized regression coefficients, the pursuit of sports community culture, the pursuit of sports health knowledge and skills, the motivation to enhance sports health knowledge and skills, the behavior of acquiring sports health knowledge and skills, as well as the 13 respective dimensions and 57 sub-factors. The C.R. value ranges from 5.496 to 13.177, with p<0.001. S.E value ranges from 0.009 to 0.147. All external variables exhibit non-negative errors, and there are no violations of the fundamental mode fit test criteria, the path has statistical significance, thereby conforming to statistical standards.

**Table 3:** Factor fit in the SEM.

Path	Estimate	S.E.	C.R.	Error	Estimate	S.E.	C.R.
C1←C	1			e1	0.356	0.031	11.465***
C2←C	1.141	0.099	11.546***	e2	0.117	0.009	12.651***
A3←A	1			e3	0.1	0.009	11.597***
A2←A	0.903	0.072	12.559***	e4	0.106	0.009	11.294***
A1←A	1.934	0.147	13.177***	e5	0.129	0.012	10.806***
A4←A	1.087	0.081	13.458***	e6	0.153	0.017	8.934***
B3←B	1			e7	0.167	0.013	12.873***
B2←B	1.591	0.144	11.041***	e8	0.222	0.019	11.726***
B1←B	1.195	0.107	11.122***	e9	0.127	0.017	7.677***
D1←D	1			e10	0.211	0.017	12.517***
D2←D	1.051	0.89	11.783***	e11	0.143	0.013	10.813***
D3←D	1.171	0.101	11.546***	e12	0.182	0.017	10.976***
D4←D	1.013	0.090	11.310***	e13	0.166	0.014	11.679***
				e14	0.049	0.009	5.496***
				e15	0.076	0.014	5.498***
				e16	0.094	0.015	6.285***

Note

*p<0.5

**p<0.01

***p<0.001.

Community Culture (A): A1-4: Community Attraction (A1), Community Norms (A2), Community Maintenance (A3), and Social Capital (A4); Pursuit of Sports Health Knowledge and Skills (B): B1-4: Self-Efficacy (B1), Self-Enrichment (B2), Self-Presentation (B3); Motivation to enhance Sports Health Knowledge and Skills (C): Discrepancy Gap in Sports Health Knowledge and Skills (C1); Enjoyment Motivation to New Sports Health Knowledge and Skills (C2); Behavior of Acquiring Sports Health Knowledge and Skills (D): Behavioral Involvement (D1), Cognitive Involvement (D2), Emotional Involvement (D3), and Socio-psychological Involvement (D4).

### 3.4 The analysis of the fit in the Structural Equation Modeling (SEM)

Regarding structural validity, a value of 1 < χ2/df < 3 is considered acceptable (lower values being more preferable). The values of NFI, GFI, AGFI, GFI, IFI, RFI, and CFI are generally higher than 0.9 (higher values being preferable). The values of PGFI, PNFI, and PCFI are generally higher than 0.5 (higher values being preferable). The value of RMSEA is generally less than 0.1 (a lower value being preferable). The value of RMR is generally less than 0.05 (a lower value being preferable). From Table 4, it can be inferred that the mode fit indices and construct validity in this study are satisfactory.

**Table 4:** The analysis of the fitting in the SEM.

χ2/df	NFI	GFI	RMSEA	RMR	CFI	IFI	RFI	CFI	PGFI	PNFI	PCFI
1.769	0.947	0.965	0.041	0.014	0.967	0.976	0.932	0.976	0.647	0.740	0.763

### 3.5 The analysis of path relationships in the SEM

**Fig 1:**
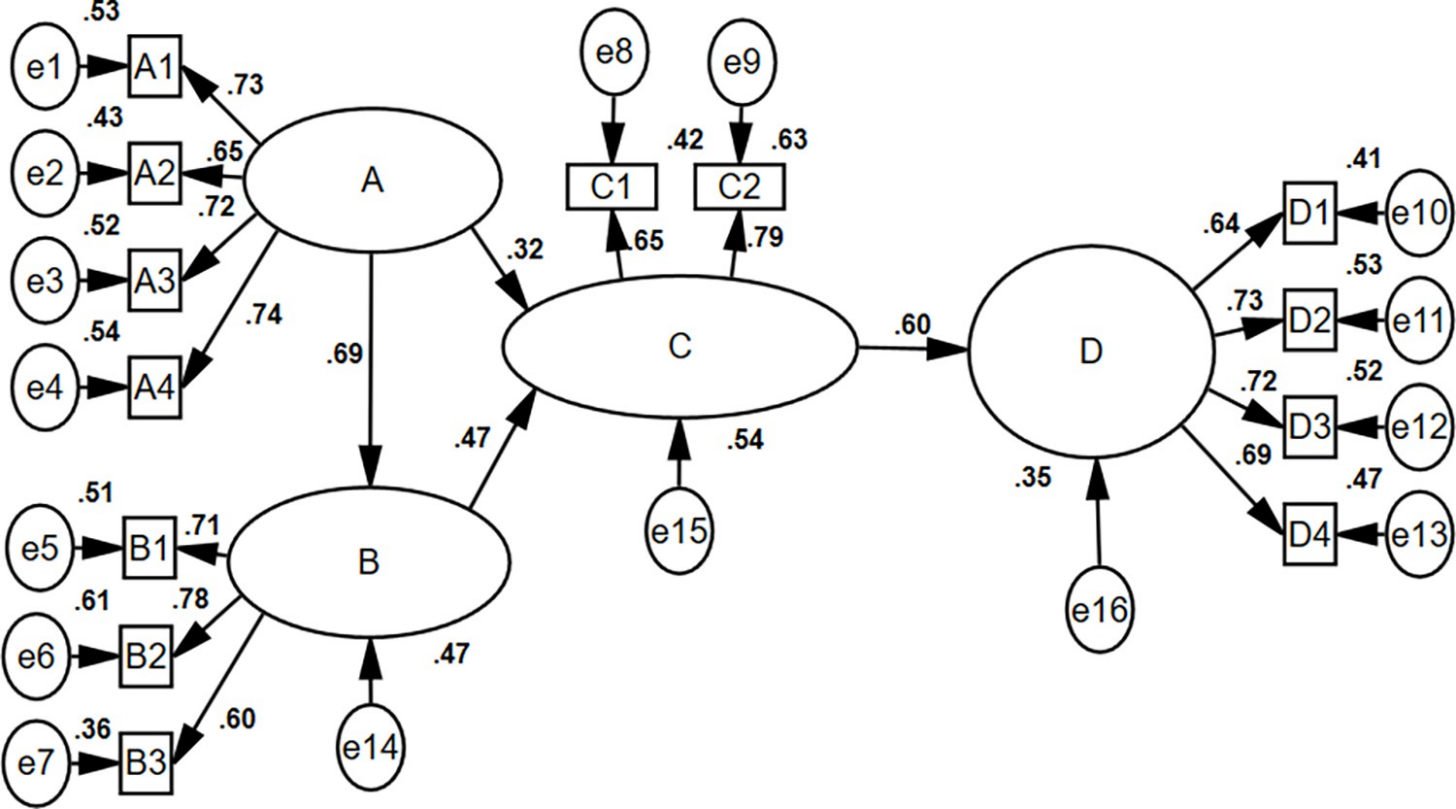
The analysis of path relationships in the SEM.

Fig 1 and Table 5 The influence of older adults sports community culture on the pursuit of sports health knowledge and skills is statistically significant (*β* = 0.69; *P*<0.001); this indicates that the more positive older adults sports community culture is, the stronger the driving force towards the pursuit of sports health knowledge and skills shows. The influence of older adults sports community culture on the motivation to enhance sports health knowledge and skills is statistically significant (*β* = 0.32, *P*<0.001); this indicates that the more positive older adults sports community culture, the stronger the motivation to enhance sports health knowledge and skills. The influence of the pursuit of sports health knowledge and skills on the motivation to enhance sports health knowledge and skills is statistically significant (*β* = 0.47, *P*<0.001); this indicates that the stronger the driving force towards the pursuit of sports health knowledge and skills among older people, the stronger their motivation to enhance such knowledge and skills. The influence of the motivation to enhance sports health knowledge and skills on the behavior of acquiring such knowledge and skills is statistically significant (*β* = 0.60; *P*<0.001); this indicates that the stronger the motivation to enhance sports health knowledge and skills among older people, the more effective their behavior in acquiring such knowledge and skills. The motivation to enhance sports health knowledge and skills is a mediating variable in Path 1: sports community culture → pursuit of sports health knowledge and skills → motivation to enhance sports health knowledge and skills (*β* = 0.192, *P*<0.001). The pursuit of sports health knowledge and skills, as well as the motivation to improve sports health knowledge and skills, is mediating variables in Path 2 (*β* = 0.195; *P*<0.001): sports community culture → motivation to enhance sports health knowledge and skills → behavior of acquiring sports health knowledge and skills, with both the pursuit of sports health knowledge and skills and the motivation to enhance such knowledge and skills acting as mediating variables.

**Table 5:** Path coefficients in the SEM.

Path	Standard path	Estimate	S.E.	C.R.	Indirect effects	Total effect	Decision
B←A(H1)	0.69	0.642	0.072	8.942***			Supported
C←A(H2)	0.32	0.402	0.105	3.817***			Supported
C←B(H3)	0.47	0.629	0.130	4.828***			Supported
C→D(H4)	0.60	0.561	0.070	8.020***			Supported
D←A(H5)					0.192***/0.195***	0.387***	Supported

Note

***P<0.001

**P<0.01

*P<0.05

## 4. Discussion

### 4.1 The Analysis of the influence of sports community culture on the pursuit of sports health knowledge and skills

The influence of sports community culture on the pursuit of sports health knowledge and skills is statistically significant (*β* = 0.69, *P*<0.001); this indicates that sports community culture has a positive impact on the pursuit of health knowledge and skills among sports community members. It also reveals that community members are deeply influenced by social attraction, social capital, community norms, and community maintenance inherent in their community organization and members. Under the influence of these factors, older adults’ willingness to enhance their sports health knowledge and skills is stimulated and achieved through self-efficacy, self-enrichment, and self-presentation. Some scholars have discussed the importance of community culture in quantitative research on personal health information management, pointing out that communal settings can stimulate a sense of competition among participants, making them more willing to change themselves and strive to achieve personal health goals [21]. Dai [30] has also proposed in their research that the sports community culture can generate a self-promotion intention toward sports health knowledge and skills among community members. This stems that sports community members have to possess a wealth of health knowledge and sports skills to align with the development of the community and their professional growth. According to the interview, sports community members will strive to enhance their sports health knowledge and skills to contribute to the development of the communities, making them more attractive and cohesive. Throughout community activities, participants often share their sports and health knowledge and athletic skills through language or actions, or they learn from one another, thereby achieving self-presentation or self-enrichment. Additionally, part of sports community norms do not impose mandatory requirements on members’ sports health knowledge and skills. While during the course of community sports activities, participants have to align with the community’s requirements to gradually refine their sports health knowledge and skills. This perspective serves as both a subjective and objective motivation for the self-professional growth of older adults. According to research findings, participants in sports communities engage in activities characterized by cooperation, fostering an environment conducive to care, enjoyment, and exchange of experience. This process also serves as a way of disseminating health knowledge and sports skills, contributing to individuals’ pursuit of sports health knowledge and skills. Drawing upon Social Comparison Theory, it can be inferred that individuals engaging in community activities generally prefer to compare themselves with those perceived as excellent. Primarily, this tendency involves comparing one’s own sports skills, viewpoints, and physical health condition with those of others, thereby stimulating insights into differences between oneself and the environment to foster a self-improvement intention.

### 4.2. The analysis of the influence of sports community culture on motivation to enhance sports health knowledge and skills

The influence of sports community culture on the motivation to enhance sports health knowledge and skills is statistically significant (*β* = 0.32, *p*<0.001), this indicates that the motivation of sports community participants to improve sports health knowledge and skills is deeply influenced by community culture. The motivation to acquire novel sports health knowledge and skills and the sense of gap in sports health knowledge and skills are significant factors in driving their motivation. These two factors are influenced by community norms, social capital, community attraction, and community maintenance, indicating that participants’ motivation is susceptible to external factors. As Social Learning Theory implies, individual observation learning is an interaction between individual behavior, intrinsic factors, and the environment. The change in personal behavior is determined by the outcome of interactions between individuals and the environment [31]. According to the research, older adults who recognize their gap in sports health knowledge or skills will be driven to pursue these knowledge and skills under the catalysis of community culture. Taking the surveyed Wushu community as an example, through communication with their peers, wushu enthusiasts who recognize their gap in wushu fitness knowledge and skills or find novel knowledge and skills in this field performed by others during activities, will be driven unconsciously to acquire these knowledge and skills. This indicates that cultural environment can trigger older adults’ motivation to acquire health knowledge and skills.

Based on the Collective Action Theory, the sustained participation of older adults in sports communities can be attributed to three factors: individual needs, the attraction of sports communities, and individual sense of responsibility. The Motivation Theory can be applied to verify this conclusion, indicating the interdependent relationship between the subject and the object [32]. Through interviews, it has been found that most older adults have a will to enhance their sports health skills. Moreover, if individuals recognize their gap in knowledge and skills during sports activities, they may develop a motivation to acquire knowledge. Furthermore, under conditions of community norms regulation, social capital support, or community attraction, community members will show more tendency to invest efforts in acquiring new knowledge and skills. All of these can contribute to achieving their sports and health goals and fulfill their professional growth. Community attraction exposes a decisive influence on the participation behavior of active social participants [33].

### 4.3 The analysis of the influence of the pursuit of sports health knowledge and skills on the motivation to enhance sports health knowledge and skills

The influence of the pursuit of sports health knowledge and skills on the motivation to enhance such knowledge and skills is statistically significant (*β* = 0.47, *P*<0.001), this indicates that the motivation of sports community participants to improve sports health knowledge and skills is deeply influenced by their pursuit of these knowledge and skills. According to the research, collective activities in sports communities are goal-oriented. Driven by the pursuit of greater pleasure, and physical and mental rewards through sports, community members have to possess corresponding sports health knowledge and skills, which in turn triggers a targeted motivation for knowledge acquisition, leading to a collective learning behavior. However, when it comes to the involvement of older adults in sports activities, their participation predominantly stems from personal preference-driven pursuit. Individual preferences determine the motivations and objectives of participants, which are reflected in efforts to bridge gaps in sports health knowledge and skills or to explore novel sports health knowledge and skills.

The objective of members from older adults sports communities in pursuing sports health knowledge and skills is a process of self-concept refinement, mutual benefit achievement among individuals, and interaction between self-pursuit and motivation. Drawing from the Self-Concept Theory, Zheng [34] elaborated the self-pursuit from three dimensions: the actual self, the projected self, and the ideal self. Among these, the ideal self represents an individual’s aspiration for their future in his interests. To achieve this goal, the individual is driven to change himself, thus generating a motivation for action.

Among various observed factors in the pursuit of sports health knowledge and skills, the self-enrichment factor exhibits the highest loading value (*β* = 0.78). It indicates that participants expect to learn more about sports health knowledge and skills, aiming to promote their health and achieve community sports goals. They also hope to derive enjoyment from community sports activities. Therefore, the expectations of older adults participants can stimulate positive learning motivation. The factor loading for self-efficacy is 0.71. Based on research, community sports activities contain lots of unpredictable situations that may pose various challenges and tests. Participants need to maintain a high level of curiosity and motivation to continually enhance their sports health knowledge and skills to better align with the objectives of community sports.

The collective activities in sports communities resemble a dynamic arena filled with social culture and interpersonal relationships. They serve as a platform for individuals to showcase their personalities, demonstrating their sports health knowledge and skills, from which individuals win respect or praise. They provide professional assistance to others, which is also an important driver for the pursuit of knowledge. This outcome aligns with Kirsty’s [35] research, which posits that self-presentation embodies one individual’s thoughts, feelings, emotions, and experiences. It serves as an outward manifestation of a sense of superiority, leading to a feeling of self-fulfillment and subsequently fostering higher learning motivation. Zhang [36] believes that middle-aged and older adults who have been participating in square dancing for a long time have higher empathy ability, and the frequency of exercise, duration of training, and intensity of exercise are positively correlated with empathy ability.

In summary, the motivation to enhance sports health knowledge and skills is influenced by self-efficacy, self-presentation, and self-enrichment, which are also important factors in triggering and sustaining motivational development.

### 4.4 The analysis of the influence of motivation to enhance physical health knowledge and skills on the behavior of acquiring these knowledge and skills

The influence of motivation to enhance sports health knowledge and skills on the behavior of acquiring such knowledge and skills is statistically significant (*β* = 0.60, p<0.001), indicating a strong cause-and-effect relationship between these two factors.

According to the research, the older participants who realize a gap in sports health knowledge and skills when compared with others tend to perform active learning behaviors to gain a voice in community activities. Furthermore, novel ideas of enhancing sports health knowledge and skills, as well as new sports health knowledge and skills released by the media, can induce active learning behaviors among participants. Cui [37], through analyzing the literature on the behavior of acquiring sports health knowledge and skills among football community members, found that the motivation of football community members to improve their sports skills has a positive impact on the cognitive involvement and behavioral involvement of the participants. Some scholars choose sports mental health mutual aid communities as their research subject, and find that members are actively seeking scientifically effective knowledge of sports to promote mental health. They persistently rely on the internet to sports health acquire knowledge and skills to promote mental health. This indicates that individuals who realize that their existing knowledge and skills for promoting health can not meet their self-set goals tend to strive to acquire relevant knowledge and skills to respond to the need or gap in knowledge, thus generating positive acquisition behaviors [38]. Some scholars argue that the accumulation of knowledge about sports can shape the anticipatory abilities of athletes with different extents of sports experience, and the magnitude of anticipatory ability can be evaluated through knowledge gaps. Individuals who recognize that their existing knowledge and skills fail to meet their goals tend to generate a motivation to seek more information to bridge this gap, which is called information-seeking behavior [39]. The above literature demonstrates the mechanism in which motivation influences behavior, which aligns with the results of this study, indicating that the motivation to enhance knowledge and skills influences the behavior of acquiring knowledge and skills.

Among various factors influencing the behavior of acquiring sports and health knowledge and skills, cognitive involvement manifests a higher loading value (0.73), indicating that participants in sports communities consider community sports as an important part of self-growth and self-enrichment. The loading values of the other three factors are all above 0.64, indicating that older adults hold a positive attitude toward the behavior of acquiring sports health knowledge and skills.

### 4.5 The analysis of the mediating path

The Structural Equation Modeling (SEM) reveals the quantitative relationships among various factors. The overall effect of sports community culture on the behavior of acquiring sports health knowledge and skills is 0.39. The path relationships in the SEM indicate that the pursuit of health knowledge and skills, as well as the motivation to enhance sports health knowledge and skills, serve as mediating variables for Path 1 (*β* = 0.195, *P*<0.001): sports community culture → pursuit of sports health knowledge and skills → motivation to enhance sports health knowledge and skills. The motivation to enhance sports health knowledge and skills acts as a mediating variable for Path 2 (*β* = 0.192, *P*<0.001): sports community culture → motivation to enhance sports health knowledge and skills → behavior of acquiring sports health knowledge and skills. This indicates that members of sports communities are influenced by the culture of their communities, leading to a demand for the pursuit of health knowledge and skills. This stimulates participants’ motivation to enhance their sports health knowledge and skills, thereby generating behavior of acquiring such knowledge and skills. Alternatively, members of the sports communities are influenced by the culture of their communities, which triggers participants’ motivation to enhance their sports health knowledge and skills, thereby leading to the behavior of acquiring sports health knowledge and skills. The research findings align with the Behavioral Motivation Theory [40]. It indicates that the cognitive motivation process of sports community members is influenced by factors such as value significance, target pursuit, ability, and belief. When members acknowledge the value and significance of community sports, develop a corresponding willingness, and set relevant goals, their behavior will be guided to conform to these values, willingness, and target pursuit. Motivation plays a mediating role in this cognitive process [27].

## 5. Research limitations

The research is confined to the older adults in Luoyang City and exhibits regional characteristics, leading to the limit of spreading the reach findings. The research only focuses on investigating the acquisition status of sports health knowledge among the older adults sports communities. The literature review has outlined the influence of various predisposition factors. The depth of the research is limited as it employs cross-sectional data to examine the relationships among four potential variables. It is recommended that subsequent research should adopt a longitudinal design to explore the mode of acquiring sports health knowledge and skills among the older adults sports communities. This approach would allow for a clearer analysis of the evolving interplay of variables over time, rendering the research outcomes more practically valuable.

## 6. Conclusion and recommendations

This research has constructed a mode for sports community members to acquire sports health knowledge and skills from the perspective of self-health promotion, demonstrating a good fit. Community culture, the pursuit of sports health knowledge and skills, and motivation to enhance sports health knowledge and skills have a significant influence on older adults’ behavior in acquiring sports health knowledge and skills. This indicates that a positive older adults community culture can improve older adults’ self-pursuit, thereby strengthening their motivation to seek knowledge, ultimately leading to participants’ behavior of acquiring sports health knowledge and skills. Moreover, by participating in meaningful community activities and challenges, older adults can find interests, hobbies, and life goals, thereby enhancing the meaning and fulfillment of their lives.

It is recommended that the government utilize new media to disseminate sports health knowledge and skills and encourage older adults to join sports communities. Community managers can help participants improve their pursuits by leveraging the influence of community culture, thereby reinforcing their motivation and enhancing the ability of participants to acquire sports health knowledge and skills in the process of improving older adults’ health. Members of older adults sports communities should regularly engage in the exchange and demonstration of sports health knowledge and skills to reinforce their health motivation. Sports experts can provide psychological support and health education to older adults through the Internet or by visiting older adults communities, helping them understand and manage their psychological and emotional needs.

## Supporting information

S1 File (ZIP)

## Abbreviations

SEM: Structural Equation Model
