# Supplementary material for: Self-health promotion: A study on the mode of acquiring sports health knowledge and skills among older adults members of sports communities
Source: PLoS One. 2024 Jul 11;19(7):e0304814. doi: 10.1371/journal.pone.0304814 (PMC11239043; doi:10.1371/journal.pone.0304814)
Supplement: S1 File — (ZIP) [file pone.0304814.s001.zip › data-Self-health Promotion/data.-word.docx]

**data-Self-health Promotion-word**


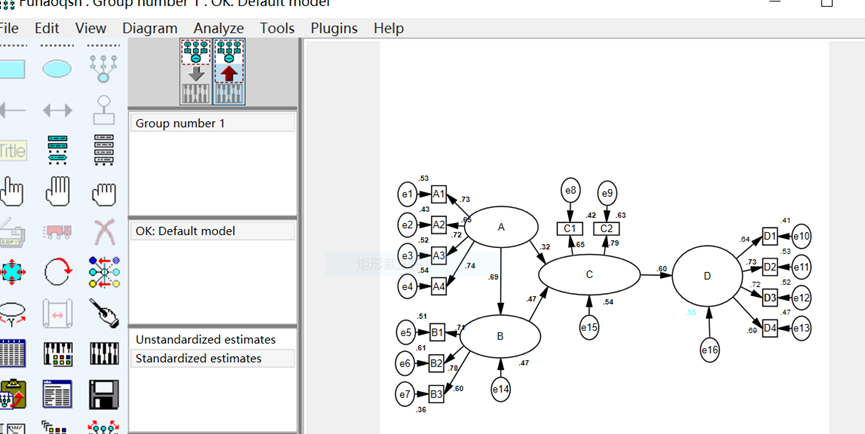


GET

FILE='C:\Users\Administrator\Desktop\健康促5.5进\Self-health Promotion\符号qsn多新健康促进.sav'.

DATASET NAME 数据集1 WINDOW=FRONT.

FREQUENCIES VARIABLES=p1 p2 p3 p4 p5 P6

/ORDER=ANALYSIS.

**频率**

| **附注** | | |
| --- | --- | --- |
| 创建的输出 | | 04-MAY-2024 09:14:41 |
| 注释 | |  |
| 输入 | 数据 | C:\Users\Administrator\Desktop\健康促5.5进\Self-health Promotion\符号qsn多新健康促进.sav |
|  | 活动的数据集 | 数据集1 |
|  | 过滤器 | <none> |
|  | 权重 | <none> |
|  | 拆分文件 | <none> |
|  | 工作数据文件中的 N 行 | 457 |
| 缺失值处理 | 对缺失的定义 | 用户定义的丢失值作为丢失对待。 |
|  | 使用的案例 | 统计量的计算将基于所有包含有效数据的案例。 |
| 语法 | | FREQUENCIES VARIABLES=p1 p2 p3 p4 p5 P6  /ORDER=ANALYSIS. |
| 资源 | 处理器时间 | 00:00:00.02 |
|  | 已用时间 | 00:00:00.02 |

[数据集1] C:\Users\Administrator\Desktop\健康促5.5进\Self-health Promotion\符号qsn多新健康促进.sav

| **统计量** | | | | | | | |
| --- | --- | --- | --- | --- | --- | --- | --- |
|  | | p1 | p2 | p3 | p4 | p5 | P6 |
| N | 有效 | 457 | 457 | 457 | 457 | 0 | 457 |
|  | 缺失 | 0 | 0 | 0 | 0 | 457 | 0 |

**频率表**

| **p1** | | | | | |
| --- | --- | --- | --- | --- | --- |
|  | | 频率 | 百分比 | 有效百分比 | 累积百分比 |
| 有效 | 1 | 166 | 36.3 | 36.3 | 36.3 |
|  | 2 | 291 | 63.7 | 63.7 | 100.0 |
|  | 合计 | 457 | 100.0 | 100.0 |  |

| **p2** | | | | | |
| --- | --- | --- | --- | --- | --- |
|  | | 频率 | 百分比 | 有效百分比 | 累积百分比 |
| 有效 | 1.00 | 79 | 17.3 | 17.3 | 17.3 |
|  | 2.00 | 130 | 28.4 | 28.4 | 45.7 |
|  | 3.00 | 134 | 29.3 | 29.3 | 75.1 |
|  | 4.00 | 114 | 24.9 | 24.9 | 100.0 |
|  | 合计 | 457 | 100.0 | 100.0 |  |

| **p3** | | | | | |
| --- | --- | --- | --- | --- | --- |
|  | | 频率 | 百分比 | 有效百分比 | 累积百分比 |
| 有效 | 1.00 | 63 | 13.8 | 13.8 | 13.8 |
|  | 2.00 | 157 | 34.4 | 34.4 | 48.1 |
|  | 3.00 | 237 | 51.9 | 51.9 | 100.0 |
|  | 合计 | 457 | 100.0 | 100.0 |  |

| **p4** | | | | | |
| --- | --- | --- | --- | --- | --- |
|  | | 频率 | 百分比 | 有效百分比 | 累积百分比 |
| 有效 | 1.00 | 238 | 52.1 | 52.1 | 52.1 |
|  | 2.00 | 219 | 47.9 | 47.9 | 100.0 |
|  | 合计 | 457 | 100.0 | 100.0 |  |

| **p5** | | | |
| --- | --- | --- | --- |
|  | | 频率 | 百分比 |
| 缺失 | 系统 | 457 | 100.0 |

| **P6** | | | | | |
| --- | --- | --- | --- | --- | --- |
|  | | 频率 | 百分比 | 有效百分比 | 累积百分比 |
| 有效 | 1.00 | 127 | 27.8 | 27.8 | 27.8 |
|  | 2.00 | 113 | 24.7 | 24.7 | 52.5 |
|  | 3.00 | 117 | 25.6 | 25.6 | 78.1 |
|  | 4.00 | 100 | 21.9 | 21.9 | 100.0 |
|  | 合计 | 457 | 100.0 | 100.0 |  |

DESCRIPTIVES VARIABLES=D11 D12 D13 D14 D15 D21 D22 D23 D24 D31 D32 D33 D34 D41 D42 D43 D44 A11 A12 A13 A14 A21 A22 A23 A24 A31 A32 A33 A34 A41 A42 A43 A44 B11 B12 B13 B14 B15 B21 B22 B23 B24 B31 B32 B33 B34 C11 C12 C13 C14 C15 C16 C21 C22 C23 C24 C25 C26

/STATISTICS=MEAN STDDEV KURTOSIS SKEWNESS.

**描述**

| **附注** | | |
| --- | --- | --- |
| 创建的输出 | | 04-MAY-2024 09:17:20 |
| 注释 | |  |
| 输入 | 数据 | C:\Users\Administrator\Desktop\健康促5.5进\Self-health Promotion\符号qsn多新健康促进.sav |
|  | 活动的数据集 | 数据集1 |
|  | 过滤器 | <none> |
|  | 权重 | <none> |
|  | 拆分文件 | <none> |
|  | 工作数据文件中的 N 行 | 457 |
| 缺失值处理 | 对缺失的定义 | 用户定义的缺失值作为缺失数据对待。 |
|  | 使用的案例 | 使用所有非缺失数据。 |
| 语法 | | DESCRIPTIVES VARIABLES=D11 D12 D13 D14 D15 D21 D22 D23 D24 D31 D32 D33 D34 D41 D42 D43 D44 A11 A12 A13 A14 A21 A22 A23 A24 A31 A32 A33 A34 A41 A42 A43 A44 B11 B12 B13 B14 B15 B21 B22 B23 B24 B31 B32 B33 B34 C11 C12 C13 C14 C15 C16 C21 C22 C23 C24 C25 C26  /STATISTICS=MEAN STDDEV KURTOSIS SKEWNESS. |
| 资源 | 处理器时间 | 00:00:00.02 |
|  | 已用时间 | 00:00:00.02 |

[数据集1] C:\Users\Administrator\Desktop\健康促5.5进\Self-health Promotion\符号qsn多新健康促进.sav

| **描述统计量** | | | | | | | |
| --- | --- | --- | --- | --- | --- | --- | --- |
|  | N | 均值 | 标准差 | 偏度 | | 峰度 | |
|  | 统计量 | 统计量 | 统计量 | 统计量 | 标准误 | 统计量 | 标准误 |
| D11 | 457 | 4.04 | .738 | -.749 | .114 | .870 | .228 |
| D12 | 457 | 4.02 | .899 | -.774 | .114 | -.055 | .228 |
| D13 | 457 | 3.99 | .759 | -.616 | .114 | .385 | .228 |
| D14 | 457 | 4.42 | .702 | -1.166 | .114 | 1.380 | .228 |
| D15 | 457 | 3.97 | .855 | -.807 | .114 | .286 | .228 |
| D21 | 457 | 3.58 | .718 | -.191 | .114 | .221 | .228 |
| D22 | 457 | 3.57 | .778 | -.624 | .114 | .832 | .228 |
| D23 | 457 | 3.45 | .751 | -.276 | .114 | .438 | .228 |
| D24 | 457 | 3.54 | .740 | -.230 | .114 | .102 | .228 |
| D31 | 457 | 3.60 | .760 | -.189 | .114 | -.113 | .228 |
| D32 | 457 | 3.41 | .817 | -.292 | .114 | .168 | .228 |
| D33 | 457 | 3.40 | .806 | -.174 | .114 | .170 | .228 |
| D34 | 457 | 3.58 | .821 | -.576 | .114 | .514 | .228 |
| D41 | 457 | 3.74 | .701 | -.166 | .114 | -.122 | .228 |
| D42 | 457 | 3.66 | .739 | -.378 | .114 | .501 | .228 |
| D43 | 457 | 3.76 | .729 | -.308 | .114 | -.013 | .228 |
| D44 | 457 | 3.60 | .760 | -.129 | .114 | -.307 | .228 |
| A11 | 457 | 3.65 | 1.046 | -.604 | .114 | -.246 | .228 |
| A12 | 457 | 3.72 | .979 | -.579 | .114 | -.183 | .228 |
| A13 | 457 | 3.38 | 1.122 | -.201 | .114 | -.792 | .228 |
| A14 | 457 | 3.53 | 1.026 | -.331 | .114 | -.611 | .228 |
| A21 | 457 | 3.91 | .539 | -.322 | .114 | 1.135 | .228 |
| A22 | 457 | 3.77 | .659 | -.555 | .114 | 1.216 | .228 |
| A23 | 457 | 4.04 | .591 | -.266 | .114 | .822 | .228 |
| A24 | 457 | 4.16 | .602 | -.142 | .114 | -.106 | .228 |
| A31 | 457 | 3.92 | .527 | -.452 | .114 | 1.653 | .228 |
| A32 | 457 | 3.94 | .555 | -.027 | .114 | .228 | .228 |
| A33 | 457 | 3.91 | .629 | -.038 | .114 | -.209 | .228 |
| A34 | 457 | 4.06 | .723 | -.331 | .114 | -.321 | .228 |
| A41 | 457 | 3.69 | .810 | -.207 | .114 | -.415 | .228 |
| A42 | 457 | 3.99 | .649 | -.427 | .114 | 1.319 | .228 |
| A43 | 457 | 4.11 | .558 | .036 | .114 | .084 | .228 |
| A44 | 457 | 3.97 | .555 | -.169 | .114 | .780 | .228 |
| B11 | 457 | 4.03 | .595 | -.134 | .114 | .284 | .228 |
| B12 | 457 | 3.83 | .655 | .005 | .114 | -.331 | .228 |
| B13 | 457 | 3.87 | .682 | -.204 | .114 | .209 | .228 |
| B14 | 457 | 3.74 | .661 | -.073 | .114 | -.162 | .228 |
| B15 | 457 | 3.53 | .749 | .055 | .114 | .009 | .228 |
| B21 | 457 | 3.31 | .790 | -.080 | .114 | .205 | .228 |
| B22 | 457 | 3.34 | .833 | -.208 | .114 | -.142 | .228 |
| B23 | 457 | 3.46 | .791 | -.233 | .114 | .193 | .228 |
| B24 | 457 | 3.46 | .740 | -.147 | .114 | .198 | .228 |
| B31 | 457 | 3.50 | .741 | -.191 | .114 | -.125 | .228 |
| B32 | 457 | 3.74 | .730 | -.394 | .114 | .275 | .228 |
| B33 | 457 | 3.60 | .746 | -.209 | .114 | -.057 | .228 |
| B34 | 457 | 3.63 | .735 | -.531 | .114 | .236 | .228 |
| C11 | 457 | 4.07 | .735 | -.546 | .114 | .397 | .228 |
| C12 | 457 | 4.13 | .751 | -.661 | .114 | .630 | .228 |
| C13 | 457 | 4.07 | .722 | -.520 | .114 | .448 | .228 |
| C14 | 457 | 4.15 | .733 | -.482 | .114 | -.269 | .228 |
| C15 | 457 | 4.00 | .806 | -.674 | .114 | .741 | .228 |
| C16 | 457 | 4.11 | .753 | -.523 | .114 | -.104 | .228 |
| C21 | 457 | 4.07 | .761 | -.539 | .114 | .011 | .228 |
| C22 | 457 | 4.04 | .822 | -.663 | .114 | .048 | .228 |
| C23 | 457 | 4.25 | .712 | -.765 | .114 | .584 | .228 |
| C24 | 457 | 4.32 | .681 | -.672 | .114 | .019 | .228 |
| C25 | 457 | 4.08 | .801 | -.767 | .114 | .382 | .228 |
| C26 | 457 | 4.13 | .721 | -.382 | .114 | -.432 | .228 |
| 有效的 N （列表状态） | 457 |  |  |  |  |  |  |

RELIABILITY

/VARIABLES=A11 A12 A13 A14 A21 A22 A23 A24 A31 A32 A33 A34 A41 A42 A43 A44

/SCALE('ALL VARIABLES') ALL

/MODEL=ALPHA.

**可靠性**

| **附注** | | |
| --- | --- | --- |
| 创建的输出 | | 04-MAY-2024 09:17:53 |
| 注释 | |  |
| 输入 | 数据 | C:\Users\Administrator\Desktop\健康促5.5进\Self-health Promotion\符号qsn多新健康促进.sav |
|  | 活动的数据集 | 数据集1 |
|  | 过滤器 | <none> |
|  | 权重 | <none> |
|  | 拆分文件 | <none> |
|  | 工作数据文件中的 N 行 | 457 |
|  | 矩阵输入 |  |
| 缺失值处理 | 对缺失的定义 | 用户定义的缺失值作为缺失数据对待。 |
|  | 使用的案例 | 统计量的计算将基于带有有效数据的所有案例，而这些有效数据适用于程序中的所有变量。 |
| 语法 | | RELIABILITY  /VARIABLES=A11 A12 A13 A14 A21 A22 A23 A24 A31 A32 A33 A34 A41 A42 A43 A44  /SCALE('ALL VARIABLES') ALL  /MODEL=ALPHA. |
| 资源 | 处理器时间 | 00:00:00.00 |
|  | 已用时间 | 00:00:00.00 |

[数据集1] C:\Users\Administrator\Desktop\健康促5.5进\Self-health Promotion\符号qsn多新健康促进.sav

**标度:所有变量**

| **案例处理汇总** | | | |
| --- | --- | --- | --- |
|  | | N | % |
| 案例 | 有效 | 457 | 100.0 |
|  | 已排除^a^ | 0 | .0 |
|  | 总计 | 457 | 100.0 |
| a. 在此程序中基于所有变量的列表方式删除。 | | | |

| **可靠性统计量** | |
| --- | --- |
| Cronbach's Alpha | 项数 |
| .885 | 16 |

RELIABILITY

/VARIABLES=B11 B12 B13 B14 B15 B21 B22 B23 B24 B31 B32 B33 B34

/SCALE('ALL VARIABLES') ALL

/MODEL=ALPHA.

**可靠性**

| **附注** | | |
| --- | --- | --- |
| 创建的输出 | | 04-MAY-2024 09:18:28 |
| 注释 | |  |
| 输入 | 数据 | C:\Users\Administrator\Desktop\健康促5.5进\Self-health Promotion\符号qsn多新健康促进.sav |
|  | 活动的数据集 | 数据集1 |
|  | 过滤器 | <none> |
|  | 权重 | <none> |
|  | 拆分文件 | <none> |
|  | 工作数据文件中的 N 行 | 457 |
|  | 矩阵输入 |  |
| 缺失值处理 | 对缺失的定义 | 用户定义的缺失值作为缺失数据对待。 |
|  | 使用的案例 | 统计量的计算将基于带有有效数据的所有案例，而这些有效数据适用于程序中的所有变量。 |
| 语法 | | RELIABILITY  /VARIABLES=B11 B12 B13 B14 B15 B21 B22 B23 B24 B31 B32 B33 B34  /SCALE('ALL VARIABLES') ALL  /MODEL=ALPHA. |
| 资源 | 处理器时间 | 00:00:00.00 |
|  | 已用时间 | 00:00:00.00 |

[数据集1] C:\Users\Administrator\Desktop\健康促5.5进\Self-health Promotion\符号qsn多新健康促进.sav

**标度:所有变量**

| **案例处理汇总** | | | |
| --- | --- | --- | --- |
|  | | N | % |
| 案例 | 有效 | 457 | 100.0 |
|  | 已排除^a^ | 0 | .0 |
|  | 总计 | 457 | 100.0 |
| a. 在此程序中基于所有变量的列表方式删除。 | | | |

| **可靠性统计量** | |
| --- | --- |
| Cronbach's Alpha | 项数 |
| .864 | 13 |

RELIABILITY

/VARIABLES=C11 C12 C13 C14 C15 C16 C21 C22 C23 C24 C25 C26

/SCALE('ALL VARIABLES') ALL

/MODEL=ALPHA.

**可靠性**

| **附注** | | |
| --- | --- | --- |
| 创建的输出 | | 04-MAY-2024 09:18:45 |
| 注释 | |  |
| 输入 | 数据 | C:\Users\Administrator\Desktop\健康促5.5进\Self-health Promotion\符号qsn多新健康促进.sav |
|  | 活动的数据集 | 数据集1 |
|  | 过滤器 | <none> |
|  | 权重 | <none> |
|  | 拆分文件 | <none> |
|  | 工作数据文件中的 N 行 | 457 |
|  | 矩阵输入 |  |
| 缺失值处理 | 对缺失的定义 | 用户定义的缺失值作为缺失数据对待。 |
|  | 使用的案例 | 统计量的计算将基于带有有效数据的所有案例，而这些有效数据适用于程序中的所有变量。 |
| 语法 | | RELIABILITY  /VARIABLES=C11 C12 C13 C14 C15 C16 C21 C22 C23 C24 C25 C26  /SCALE('ALL VARIABLES') ALL  /MODEL=ALPHA. |
| 资源 | 处理器时间 | 00:00:00.00 |
|  | 已用时间 | 00:00:00.01 |

[数据集1] C:\Users\Administrator\Desktop\健康促5.5进\Self-health Promotion\符号qsn多新健康促进.sav

**标度:所有变量**

| **案例处理汇总** | | | |
| --- | --- | --- | --- |
|  | | N | % |
| 案例 | 有效 | 457 | 100.0 |
|  | 已排除^a^ | 0 | .0 |
|  | 总计 | 457 | 100.0 |
| a. 在此程序中基于所有变量的列表方式删除。 | | | |

| **可靠性统计量** | |
| --- | --- |
| Cronbach's Alpha | 项数 |
| .908 | 12 |

RELIABILITY

/VARIABLES=D11 D12 D13 D14 D15 D21 D22 D23 D24 D31 D32 D33 D34 D41 D42 D43 D44

/SCALE('ALL VARIABLES') ALL

/MODEL=ALPHA.

**可靠性**

| **附注** | | |
| --- | --- | --- |
| 创建的输出 | | 04-MAY-2024 09:20:42 |
| 注释 | |  |
| 输入 | 数据 | C:\Users\Administrator\Desktop\健康促5.5进\Self-health Promotion\符号qsn多新健康促进.sav |
|  | 活动的数据集 | 数据集1 |
|  | 过滤器 | <none> |
|  | 权重 | <none> |
|  | 拆分文件 | <none> |
|  | 工作数据文件中的 N 行 | 457 |
|  | 矩阵输入 |  |
| 缺失值处理 | 对缺失的定义 | 用户定义的缺失值作为缺失数据对待。 |
|  | 使用的案例 | 统计量的计算将基于带有有效数据的所有案例，而这些有效数据适用于程序中的所有变量。 |
| 语法 | | RELIABILITY  /VARIABLES=D11 D12 D13 D14 D15 D21 D22 D23 D24 D31 D32 D33 D34 D41 D42 D43 D44  /SCALE('ALL VARIABLES') ALL  /MODEL=ALPHA. |
| 资源 | 处理器时间 | 00:00:00.00 |
|  | 已用时间 | 00:00:00.00 |

[数据集1] C:\Users\Administrator\Desktop\健康促5.5进\Self-health Promotion\符号qsn多新健康促进.sav

**标度:所有变量**

| **案例处理汇总** | | | |
| --- | --- | --- | --- |
|  | | N | % |
| 案例 | 有效 | 457 | 100.0 |
|  | 已排除^a^ | 0 | .0 |
|  | 总计 | 457 | 100.0 |
| a. 在此程序中基于所有变量的列表方式删除。 | | | |

| **可靠性统计量** | |
| --- | --- |
| Cronbach's Alpha | 项数 |
| .900 | 17 |

FACTOR

/VARIABLES D11 D12 D13 D14 D15 D21 D22 D23 D24 D31 D32 D33 D34 D41 D42 D43 D44

/MISSING LISTWISE

/ANALYSIS D11 D12 D13 D14 D15 D21 D22 D23 D24 D31 D32 D33 D34 D41 D42 D43 D44

/PRINT INITIAL KMO EXTRACTION ROTATION

/FORMAT BLANK(.5)

/CRITERIA FACTORS(4) ITERATE(25)

/EXTRACTION PC

/CRITERIA ITERATE(25)

/ROTATION VARIMAX

/METHOD=CORRELATION.

**因子分析**

| **附注** | | |
| --- | --- | --- |
| 创建的输出 | | 04-MAY-2024 09:24:25 |
| 注释 | |  |
| 输入 | 数据 | C:\Users\Administrator\Desktop\健康促5.5进\Self-health Promotion\符号qsn多新健康促进.sav |
|  | 活动的数据集 | 数据集1 |
|  | 过滤器 | <none> |
|  | 权重 | <none> |
|  | 拆分文件 | <none> |
|  | 工作数据文件中的 N 行 | 457 |
| 缺失值处理 | 对缺失的定义 | MISSING=EXCLUDE：用户定义的缺失值作为缺失对待。 |
|  | 使用的案例 | LISTWISE：统计量基于对所使用任何变量都不含缺失值的案例。 |
| 语法 | | FACTOR  /VARIABLES D11 D12 D13 D14 D15 D21 D22 D23 D24 D31 D32 D33 D34 D41 D42 D43 D44  /MISSING LISTWISE  /ANALYSIS D11 D12 D13 D14 D15 D21 D22 D23 D24 D31 D32 D33 D34 D41 D42 D43 D44  /PRINT INITIAL KMO EXTRACTION ROTATION  /FORMAT BLANK(.5)  /CRITERIA FACTORS(4) ITERATE(25)  /EXTRACTION PC  /CRITERIA ITERATE(25)  /ROTATION VARIMAX  /METHOD=CORRELATION. |
| 资源 | 处理器时间 | 00:00:00.00 |
|  | 已用时间 | 00:00:00.00 |
|  | 所需的最大内存 | 35684 (34.848K) 字节 |

[数据集1] C:\Users\Administrator\Desktop\健康促5.5进\Self-health Promotion\符号qsn多新健康促进.sav

| **KMO 和 Bartlett 的检验** | | |
| --- | --- | --- |
| 取样足够度的 Kaiser-Meyer-Olkin 度量。 | | .914 |
| Bartlett 的球形度检验 | 近似卡方 | 3129.508 |
|  | df | 136 |
|  | Sig. | .000 |

| **公因子方差** | | |
| --- | --- | --- |
|  | 初始 | 提取 |
| D11 | 1.000 | .767 |
| D12 | 1.000 | .480 |
| D13 | 1.000 | .704 |
| D14 | 1.000 | .444 |
| D15 | 1.000 | .776 |
| D21 | 1.000 | .596 |
| D22 | 1.000 | .560 |
| D23 | 1.000 | .661 |
| D24 | 1.000 | .479 |
| D31 | 1.000 | .544 |
| D32 | 1.000 | .619 |
| D33 | 1.000 | .740 |
| D34 | 1.000 | .610 |
| D41 | 1.000 | .652 |
| D42 | 1.000 | .652 |
| D43 | 1.000 | .705 |
| D44 | 1.000 | .529 |
| 提取方法：主成份分析。 | | |

| **解释的总方差** | | | | | | | | | |
| --- | --- | --- | --- | --- | --- | --- | --- | --- | --- |
| 成份 | 初始特征值 | | | 提取平方和载入 | | | 旋转平方和载入 | | |
|  | 合计 | 方差的 % | 累积 % | 合计 | 方差的 % | 累积 % | 合计 | 方差的 % | 累积 % |
| 1 | 6.582 | 38.720 | 38.720 | 6.582 | 38.720 | 38.720 | 2.949 | 17.349 | 17.349 |
| 2 | 1.555 | 9.146 | 47.866 | 1.555 | 9.146 | 47.866 | 2.601 | 15.302 | 32.651 |
| 3 | 1.221 | 7.181 | 55.046 | 1.221 | 7.181 | 55.046 | 2.537 | 14.923 | 47.574 |
| 4 | 1.092 | 6.421 | 61.467 | 1.092 | 6.421 | 61.467 | 2.362 | 13.892 | 61.467 |
| 5 | .854 | 5.024 | 66.491 |  |  |  |  |  |  |
| 6 | .680 | 4.000 | 70.491 |  |  |  |  |  |  |
| 7 | .640 | 3.764 | 74.256 |  |  |  |  |  |  |
| 8 | .588 | 3.458 | 77.714 |  |  |  |  |  |  |
| 9 | .541 | 3.181 | 80.894 |  |  |  |  |  |  |
| 10 | .530 | 3.116 | 84.010 |  |  |  |  |  |  |
| 11 | .484 | 2.848 | 86.857 |  |  |  |  |  |  |
| 12 | .456 | 2.685 | 89.542 |  |  |  |  |  |  |
| 13 | .442 | 2.599 | 92.141 |  |  |  |  |  |  |
| 14 | .401 | 2.360 | 94.501 |  |  |  |  |  |  |
| 15 | .372 | 2.186 | 96.687 |  |  |  |  |  |  |
| 16 | .337 | 1.981 | 98.668 |  |  |  |  |  |  |
| 17 | .226 | 1.332 | 100.000 |  |  |  |  |  |  |
| 提取方法：主成份分析。 | | | | | | | | | |

| **成份矩阵^a^** | | | | |
| --- | --- | --- | --- | --- |
|  | 成份 | | | |
|  | 1 | 2 | 3 | 4 |
| D11 | .651 | .567 |  |  |
| D12 | .588 |  |  |  |
| D13 | .690 |  |  |  |
| D14 | .542 |  |  |  |
| D15 | .680 | .525 |  |  |
| D21 | .617 |  |  |  |
| D22 | .579 |  |  |  |
| D23 | .612 |  |  |  |
| D24 | .592 |  |  |  |
| D31 | .654 |  |  |  |
| D32 | .627 |  |  |  |
| D33 | .685 |  |  |  |
| D34 | .604 |  |  |  |
| D41 | .634 |  |  |  |
| D42 | .646 |  |  |  |
| D43 | .626 |  |  |  |
| D44 | .600 |  |  |  |
| 提取方法 :主成份。 | | | | |
| a. 已提取了 4 个成份。 | | | | |

| **旋转成份矩阵^a^** | | | | |
| --- | --- | --- | --- | --- |
|  | 成份 | | | |
|  | 1 | 2 | 3 | 4 |
| D11 | .840 |  |  |  |
| D12 | .619 |  |  |  |
| D13 | .769 |  |  |  |
| D14 | .602 |  |  |  |
| D15 | .830 |  |  |  |
| D21 |  |  |  | .686 |
| D22 |  |  |  | .674 |
| D23 |  |  |  | .760 |
| D24 |  |  |  | .569 |
| D31 |  |  | .598 |  |
| D32 |  |  | .719 |  |
| D33 |  |  | .782 |  |
| D34 |  |  | .721 |  |
| D41 |  | .735 |  |  |
| D42 |  | .742 |  |  |
| D43 |  | .793 |  |  |
| D44 |  | .637 |  |  |
| 提取方法 :主成份。  旋转法 :具有 Kaiser 标准化的正交旋转法。^a^ | | | | |
| a. 旋转在 6 次迭代后收敛。 | | | | |

| **成份转换矩阵** | | | | |
| --- | --- | --- | --- | --- |
| 成份 | 1 | 2 | 3 | 4 |
| 1 | .529 | .498 | .500 | .471 |
| 2 | .830 | -.417 | -.352 | -.117 |
| 3 | -.146 | -.725 | .317 | .594 |
| 4 | -.122 | .230 | -.725 | .642 |
| 提取方法 :主成份。  旋转法 :具有 Kaiser 标准化的正交旋转法。 | | | | |

FACTOR

/VARIABLES A11 A12 A13 A14 A21 A22 A23 A24 A31 A32 A33 A34 A41 A42 A43 A44

/MISSING LISTWISE

/ANALYSIS A11 A12 A13 A14 A21 A22 A23 A24 A31 A32 A33 A34 A41 A42 A43 A44

/PRINT INITIAL KMO EXTRACTION ROTATION

/FORMAT BLANK(.5)

/CRITERIA FACTORS(4) ITERATE(25)

/EXTRACTION PC

/CRITERIA ITERATE(25)

/ROTATION VARIMAX

/METHOD=CORRELATION.

**因子分析**

| **附注** | | |
| --- | --- | --- |
| 创建的输出 | | 04-MAY-2024 09:25:38 |
| 注释 | |  |
| 输入 | 数据 | C:\Users\Administrator\Desktop\健康促5.5进\Self-health Promotion\符号qsn多新健康促进.sav |
|  | 活动的数据集 | 数据集1 |
|  | 过滤器 | <none> |
|  | 权重 | <none> |
|  | 拆分文件 | <none> |
|  | 工作数据文件中的 N 行 | 457 |
| 缺失值处理 | 对缺失的定义 | MISSING=EXCLUDE：用户定义的缺失值作为缺失对待。 |
|  | 使用的案例 | LISTWISE：统计量基于对所使用任何变量都不含缺失值的案例。 |
| 语法 | | FACTOR  /VARIABLES A11 A12 A13 A14 A21 A22 A23 A24 A31 A32 A33 A34 A41 A42 A43 A44  /MISSING LISTWISE  /ANALYSIS A11 A12 A13 A14 A21 A22 A23 A24 A31 A32 A33 A34 A41 A42 A43 A44  /PRINT INITIAL KMO EXTRACTION ROTATION  /FORMAT BLANK(.5)  /CRITERIA FACTORS(4) ITERATE(25)  /EXTRACTION PC  /CRITERIA ITERATE(25)  /ROTATION VARIMAX  /METHOD=CORRELATION. |
| 资源 | 处理器时间 | 00:00:00.03 |
|  | 已用时间 | 00:00:00.01 |
|  | 所需的最大内存 | 31864 (31.117K) 字节 |

[数据集1] C:\Users\Administrator\Desktop\健康促5.5进\Self-health Promotion\符号qsn多新健康促进.sav

| **KMO 和 Bartlett 的检验** | | |
| --- | --- | --- |
| 取样足够度的 Kaiser-Meyer-Olkin 度量。 | | .877 |
| Bartlett 的球形度检验 | 近似卡方 | 3003.539 |
|  | df | 120 |
|  | Sig. | .000 |

| **公因子方差** | | |
| --- | --- | --- |
|  | 初始 | 提取 |
| A11 | 1.000 | .708 |
| A12 | 1.000 | .777 |
| A13 | 1.000 | .699 |
| A14 | 1.000 | .695 |
| A21 | 1.000 | .641 |
| A22 | 1.000 | .707 |
| A23 | 1.000 | .619 |
| A24 | 1.000 | .477 |
| A31 | 1.000 | .553 |
| A32 | 1.000 | .658 |
| A33 | 1.000 | .519 |
| A34 | 1.000 | .508 |
| A41 | 1.000 | .394 |
| A42 | 1.000 | .611 |
| A43 | 1.000 | .738 |
| A44 | 1.000 | .646 |
| 提取方法：主成份分析。 | | |

| **解释的总方差** | | | | | | | | | |
| --- | --- | --- | --- | --- | --- | --- | --- | --- | --- |
| 成份 | 初始特征值 | | | 提取平方和载入 | | | 旋转平方和载入 | | |
|  | 合计 | 方差的 % | 累积 % | 合计 | 方差的 % | 累积 % | 合计 | 方差的 % | 累积 % |
| 1 | 6.123 | 38.267 | 38.267 | 6.123 | 38.267 | 38.267 | 2.852 | 17.823 | 17.823 |
| 2 | 1.591 | 9.943 | 48.210 | 1.591 | 9.943 | 48.210 | 2.483 | 15.517 | 33.340 |
| 3 | 1.132 | 7.076 | 55.286 | 1.132 | 7.076 | 55.286 | 2.325 | 14.530 | 47.869 |
| 4 | 1.035 | 6.472 | 61.758 | 1.035 | 6.472 | 61.758 | 2.222 | 13.889 | 61.758 |
| 5 | .824 | 5.151 | 66.909 |  |  |  |  |  |  |
| 6 | .817 | 5.108 | 72.016 |  |  |  |  |  |  |
| 7 | .711 | 4.444 | 76.460 |  |  |  |  |  |  |
| 8 | .608 | 3.797 | 80.257 |  |  |  |  |  |  |
| 9 | .582 | 3.635 | 83.892 |  |  |  |  |  |  |
| 10 | .537 | 3.357 | 87.249 |  |  |  |  |  |  |
| 11 | .475 | 2.968 | 90.217 |  |  |  |  |  |  |
| 12 | .415 | 2.594 | 92.811 |  |  |  |  |  |  |
| 13 | .373 | 2.331 | 95.143 |  |  |  |  |  |  |
| 14 | .314 | 1.964 | 97.107 |  |  |  |  |  |  |
| 15 | .264 | 1.648 | 98.755 |  |  |  |  |  |  |
| 16 | .199 | 1.245 | 100.000 |  |  |  |  |  |  |
| 提取方法：主成份分析。 | | | | | | | | | |

| **成份矩阵^a^** | | | | |
| --- | --- | --- | --- | --- |
|  | 成份 | | | |
|  | 1 | 2 | 3 | 4 |
| A11 | .677 |  |  |  |
| A12 | .678 |  |  |  |
| A13 | .699 |  |  |  |
| A14 | .706 |  |  |  |
| A21 | .615 |  |  |  |
| A22 | .623 |  |  |  |
| A23 | .590 |  |  |  |
| A24 | .517 |  |  |  |
| A31 | .531 |  |  |  |
| A32 | .617 |  |  |  |
| A33 | .588 |  |  |  |
| A34 | .620 |  |  |  |
| A41 | .544 |  |  |  |
| A42 | .609 |  |  |  |
| A43 | .658 |  |  |  |
| A44 | .636 |  |  |  |
| 提取方法 :主成份。 | | | | |
| a. 已提取了 4 个成份。 | | | | |

| **旋转成份矩阵^a^** | | | | |
| --- | --- | --- | --- | --- |
|  | 成份 | | | |
|  | 1 | 2 | 3 | 4 |
| A11 | .779 |  |  |  |
| A12 | .831 |  |  |  |
| A13 | .762 |  |  |  |
| A14 | .751 |  |  |  |
| A21 |  | .746 |  |  |
| A22 |  | .786 |  |  |
| A23 |  | .726 |  |  |
| A24 |  | .586 |  |  |
| A31 |  |  |  | .705 |
| A32 |  |  |  | .723 |
| A33 |  |  |  | .631 |
| A34 |  |  |  | .595 |
| A41 |  |  | .681 |  |
| A42 |  |  | .704 |  |
| A43 |  |  | .802 |  |
| A44 |  |  | .735 |  |
| 提取方法 :主成份。  旋转法 :具有 Kaiser 标准化的正交旋转法。^a^ | | | | |
| a. 旋转在 6 次迭代后收敛。 | | | | |

| **成份转换矩阵** | | | | |
| --- | --- | --- | --- | --- |
| 成份 | 1 | 2 | 3 | 4 |
| 1 | .555 | .483 | .495 | .463 |
| 2 | -.652 | .645 | -.214 | .336 |
| 3 | -.399 | -.554 | .450 | .575 |
| 4 | .329 | -.209 | -.712 | .584 |
| 提取方法 :主成份。  旋转法 :具有 Kaiser 标准化的正交旋转法。 | | | | |

FACTOR

/VARIABLES B11 B12 B13 B14 B15 B21 B22 B23 B24 B31 B32 B33 B34

/MISSING LISTWISE

/ANALYSIS B11 B12 B13 B14 B15 B21 B22 B23 B24 B31 B32 B33 B34

/PRINT INITIAL KMO EXTRACTION ROTATION

/FORMAT BLANK(.5)

/CRITERIA FACTORS(3) ITERATE(25)

/EXTRACTION PC

/CRITERIA ITERATE(25)

/ROTATION VARIMAX

/METHOD=CORRELATION.

**因子分析**

| **附注** | | |
| --- | --- | --- |
| 创建的输出 | | 04-MAY-2024 09:26:12 |
| 注释 | |  |
| 输入 | 数据 | C:\Users\Administrator\Desktop\健康促5.5进\Self-health Promotion\符号qsn多新健康促进.sav |
|  | 活动的数据集 | 数据集1 |
|  | 过滤器 | <none> |
|  | 权重 | <none> |
|  | 拆分文件 | <none> |
|  | 工作数据文件中的 N 行 | 457 |
| 缺失值处理 | 对缺失的定义 | MISSING=EXCLUDE：用户定义的缺失值作为缺失对待。 |
|  | 使用的案例 | LISTWISE：统计量基于对所使用任何变量都不含缺失值的案例。 |
| 语法 | | FACTOR  /VARIABLES B11 B12 B13 B14 B15 B21 B22 B23 B24 B31 B32 B33 B34  /MISSING LISTWISE  /ANALYSIS B11 B12 B13 B14 B15 B21 B22 B23 B24 B31 B32 B33 B34  /PRINT INITIAL KMO EXTRACTION ROTATION  /FORMAT BLANK(.5)  /CRITERIA FACTORS(3) ITERATE(25)  /EXTRACTION PC  /CRITERIA ITERATE(25)  /ROTATION VARIMAX  /METHOD=CORRELATION. |
| 资源 | 处理器时间 | 00:00:00.02 |
|  | 已用时间 | 00:00:00.02 |
|  | 所需的最大内存 | 21700 (21.191K) 字节 |

[数据集1] C:\Users\Administrator\Desktop\健康促5.5进\Self-health Promotion\符号qsn多新健康促进.sav

| **KMO 和 Bartlett 的检验** | | |
| --- | --- | --- |
| 取样足够度的 Kaiser-Meyer-Olkin 度量。 | | .889 |
| Bartlett 的球形度检验 | 近似卡方 | 2137.337 |
|  | df | 78 |
|  | Sig. | .000 |

| **公因子方差** | | |
| --- | --- | --- |
|  | 初始 | 提取 |
| B11 | 1.000 | .568 |
| B12 | 1.000 | .741 |
| B13 | 1.000 | .778 |
| B14 | 1.000 | .561 |
| B15 | 1.000 | .483 |
| B21 | 1.000 | .692 |
| B22 | 1.000 | .714 |
| B23 | 1.000 | .642 |
| B24 | 1.000 | .532 |
| B31 | 1.000 | .426 |
| B32 | 1.000 | .575 |
| B33 | 1.000 | .577 |
| B34 | 1.000 | .486 |
| 提取方法：主成份分析。 | | |

| **解释的总方差** | | | | | | | | | |
| --- | --- | --- | --- | --- | --- | --- | --- | --- | --- |
| 成份 | 初始特征值 | | | 提取平方和载入 | | | 旋转平方和载入 | | |
|  | 合计 | 方差的 % | 累积 % | 合计 | 方差的 % | 累积 % | 合计 | 方差的 % | 累积 % |
| 1 | 5.107 | 39.284 | 39.284 | 5.107 | 39.284 | 39.284 | 2.964 | 22.796 | 22.796 |
| 2 | 1.325 | 10.192 | 49.477 | 1.325 | 10.192 | 49.477 | 2.627 | 20.205 | 43.001 |
| 3 | 1.144 | 8.802 | 58.279 | 1.144 | 8.802 | 58.279 | 1.986 | 15.277 | 58.279 |
| 4 | .934 | 7.182 | 65.461 |  |  |  |  |  |  |
| 5 | .732 | 5.630 | 71.090 |  |  |  |  |  |  |
| 6 | .674 | 5.183 | 76.273 |  |  |  |  |  |  |
| 7 | .617 | 4.748 | 81.022 |  |  |  |  |  |  |
| 8 | .533 | 4.098 | 85.120 |  |  |  |  |  |  |
| 9 | .499 | 3.838 | 88.958 |  |  |  |  |  |  |
| 10 | .451 | 3.473 | 92.431 |  |  |  |  |  |  |
| 11 | .412 | 3.172 | 95.603 |  |  |  |  |  |  |
| 12 | .325 | 2.497 | 98.100 |  |  |  |  |  |  |
| 13 | .247 | 1.900 | 100.000 |  |  |  |  |  |  |
| 提取方法：主成份分析。 | | | | | | | | | |

| **成份矩阵^a^** | | | |
| --- | --- | --- | --- |
|  | 成份 | | |
|  | 1 | 2 | 3 |
| B11 | .625 |  |  |
| B12 | .729 |  |  |
| B13 | .771 |  |  |
| B14 | .669 |  |  |
| B15 | .634 |  |  |
| B21 | .655 |  |  |
| B22 | .700 |  |  |
| B23 | .658 |  |  |
| B24 | .654 |  |  |
| B31 | .509 |  |  |
| B32 | .511 |  | .531 |
| B33 | .590 |  |  |
| B34 | .511 |  |  |
| 提取方法 :主成份。 | | | |
| a. 已提取了 3 个成份。 | | | |

| **旋转成份矩阵^a^** | | | |
| --- | --- | --- | --- |
|  | 成份 | | |
|  | 1 | 2 | 3 |
| B11 | .724 |  |  |
| B12 | .825 |  |  |
| B13 | .829 |  |  |
| B14 | .689 |  |  |
| B15 | .576 |  |  |
| B21 |  | .802 |  |
| B22 |  | .797 |  |
| B23 |  | .754 |  |
| B24 |  | .627 |  |
| B31 |  |  | .701 |
| B32 |  |  | .731 |
| B33 |  |  | .692 |
| B34 |  |  | .656 |
| 提取方法 :主成份。  旋转法 :具有 Kaiser 标准化的正交旋转法。^a^ | | | |
| a. 旋转在 5 次迭代后收敛。 | | | |

| **成份转换矩阵** | | | |
| --- | --- | --- | --- |
| 成份 | 1 | 2 | 3 |
| 1 | .659 | .596 | .458 |
| 2 | -.737 | .633 | .237 |
| 3 | -.149 | -.494 | .857 |
| 提取方法 :主成份。  旋转法 :具有 Kaiser 标准化的正交旋转法。 | | | |

FACTOR

/VARIABLES C11 C12 C13 C14 C15 C16 C21 C22 C23 C24 C25 C26

/MISSING LISTWISE

/ANALYSIS C11 C12 C13 C14 C15 C16 C21 C22 C23 C24 C25 C26

/PRINT INITIAL KMO EXTRACTION ROTATION

/FORMAT BLANK(.5)

/CRITERIA FACTORS(2) ITERATE(25)

/EXTRACTION PC

/CRITERIA ITERATE(25)

/ROTATION VARIMAX

/METHOD=CORRELATION.

**因子分析**

| **附注** | | |
| --- | --- | --- |
| 创建的输出 | | 04-MAY-2024 09:26:33 |
| 注释 | |  |
| 输入 | 数据 | C:\Users\Administrator\Desktop\健康促5.5进\Self-health Promotion\符号qsn多新健康促进.sav |
|  | 活动的数据集 | 数据集1 |
|  | 过滤器 | <none> |
|  | 权重 | <none> |
|  | 拆分文件 | <none> |
|  | 工作数据文件中的 N 行 | 457 |
| 缺失值处理 | 对缺失的定义 | MISSING=EXCLUDE：用户定义的缺失值作为缺失对待。 |
|  | 使用的案例 | LISTWISE：统计量基于对所使用任何变量都不含缺失值的案例。 |
| 语法 | | FACTOR  /VARIABLES C11 C12 C13 C14 C15 C16 C21 C22 C23 C24 C25 C26  /MISSING LISTWISE  /ANALYSIS C11 C12 C13 C14 C15 C16 C21 C22 C23 C24 C25 C26  /PRINT INITIAL KMO EXTRACTION ROTATION  /FORMAT BLANK(.5)  /CRITERIA FACTORS(2) ITERATE(25)  /EXTRACTION PC  /CRITERIA ITERATE(25)  /ROTATION VARIMAX  /METHOD=CORRELATION. |
| 资源 | 处理器时间 | 00:00:00.00 |
|  | 已用时间 | 00:00:00.02 |
|  | 所需的最大内存 | 18744 (18.305K) 字节 |

[数据集1] C:\Users\Administrator\Desktop\健康促5.5进\Self-health Promotion\符号qsn多新健康促进.sav

| **KMO 和 Bartlett 的检验** | | |
| --- | --- | --- |
| 取样足够度的 Kaiser-Meyer-Olkin 度量。 | | .913 |
| Bartlett 的球形度检验 | 近似卡方 | 3183.664 |
|  | df | 66 |
|  | Sig. | .000 |

| **公因子方差** | | |
| --- | --- | --- |
|  | 初始 | 提取 |
| C11 | 1.000 | .755 |
| C12 | 1.000 | .806 |
| C13 | 1.000 | .737 |
| C14 | 1.000 | .675 |
| C15 | 1.000 | .525 |
| C16 | 1.000 | .590 |
| C21 | 1.000 | .638 |
| C22 | 1.000 | .639 |
| C23 | 1.000 | .613 |
| C24 | 1.000 | .399 |
| C25 | 1.000 | .722 |
| C26 | 1.000 | .642 |
| 提取方法：主成份分析。 | | |

| **解释的总方差** | | | | | | | | | |
| --- | --- | --- | --- | --- | --- | --- | --- | --- | --- |
| 成份 | 初始特征值 | | | 提取平方和载入 | | | 旋转平方和载入 | | |
|  | 合计 | 方差的 % | 累积 % | 合计 | 方差的 % | 累积 % | 合计 | 方差的 % | 累积 % |
| 1 | 5.997 | 49.974 | 49.974 | 5.997 | 49.974 | 49.974 | 4.054 | 33.787 | 33.787 |
| 2 | 1.743 | 14.525 | 64.499 | 1.743 | 14.525 | 64.499 | 3.685 | 30.711 | 64.499 |
| 3 | .785 | 6.543 | 71.042 |  |  |  |  |  |  |
| 4 | .676 | 5.629 | 76.672 |  |  |  |  |  |  |
| 5 | .505 | 4.209 | 80.881 |  |  |  |  |  |  |
| 6 | .435 | 3.621 | 84.502 |  |  |  |  |  |  |
| 7 | .401 | 3.342 | 87.844 |  |  |  |  |  |  |
| 8 | .398 | 3.320 | 91.164 |  |  |  |  |  |  |
| 9 | .323 | 2.694 | 93.858 |  |  |  |  |  |  |
| 10 | .303 | 2.526 | 96.384 |  |  |  |  |  |  |
| 11 | .260 | 2.165 | 98.549 |  |  |  |  |  |  |
| 12 | .174 | 1.451 | 100.000 |  |  |  |  |  |  |
| 提取方法：主成份分析。 | | | | | | | | | |

| **成份矩阵^a^** | | |
| --- | --- | --- |
|  | 成份 | |
|  | 1 | 2 |
| C11 | .766 |  |
| C12 | .794 |  |
| C13 | .769 |  |
| C14 | .747 |  |
| C15 | .645 |  |
| C16 | .726 |  |
| C21 | .683 |  |
| C22 | .689 |  |
| C23 | .656 |  |
| C24 | .592 |  |
| C25 | .698 |  |
| C26 | .691 |  |
| 提取方法 :主成份。 | | |
| a. 已提取了 2 个成份。 | | |

| **旋转成份矩阵^a^** | | |
| --- | --- | --- |
|  | 成份 | |
|  | 1 | 2 |
| C11 | .842 |  |
| C12 | .869 |  |
| C13 | .825 |  |
| C14 | .781 |  |
| C15 | .699 |  |
| C16 | .705 |  |
| C21 |  | .766 |
| C22 |  | .764 |
| C23 |  | .759 |
| C24 |  | .563 |
| C25 |  | .829 |
| C26 |  | .766 |
| 提取方法 :主成份。  旋转法 :具有 Kaiser 标准化的正交旋转法。^a^ | | |
| a. 旋转在 3 次迭代后收敛。 | | |

| **成份转换矩阵** | | |
| --- | --- | --- |
| 成份 | 1 | 2 |
| 1 | .737 | .676 |
| 2 | -.676 | .737 |
| 提取方法 :主成份。  旋转法 :具有 Kaiser 标准化的正交旋转法。 | | |

**C:\Users\Administrator\Desktop\健康促进\fuhaoqsn.amw**

**Analysis Summary**

**Date and Time**

Date: 2024年5月2日

Time: 7:12:24

**Title**

fuhaoqsn: 2024年5月2日 7:12

**Groups**

**Group number 1 (Group number 1)**

**Notes for Group (Group number 1)**

The model is recursive.

Sample size = 457

**Variable Summary (Group number 1)**

**Your model contains the following variables (Group number 1)**

Observed, endogenous variables

C1

C2

A3

A2

A1

B3

B2

B1

D1

D2

D3

D4

A4

Unobserved, endogenous variables

C

B

D

Unobserved, exogenous variables

e8

e9

A

e3

e2

e1

e7

e6

e10

e11

e12

e13

e4

e14

e15

e16

e5

**Variable counts (Group number 1)**

| **Number of variables in your model:** | 33 |
| --- | --- |
| **Number of observed variables:** | 13 |
| **Number of unobserved variables:** | 20 |
| **Number of exogenous variables:** | 17 |
| **Number of endogenous variables:** | 16 |

**Parameter Summary (Group number 1)**

|  | **Weights** | **Covariances** | **Variances** | **Means** | **Intercepts** | **Total** |
| --- | --- | --- | --- | --- | --- | --- |
| **Fixed** | 20 | 0 | 0 | 0 | 0 | 20 |
| **Labeled** | 0 | 0 | 0 | 0 | 0 | 0 |
| **Unlabeled** | 13 | 0 | 17 | 0 | 0 | 30 |
| **Total** | 33 | 0 | 17 | 0 | 0 | 50 |

**Models**

**Default model (Default model)**

**Notes for Model (Default model)**

**Computation of degrees of freedom (Default model)**

| **Number of distinct sample moments:** | 91 |
| --- | --- |
| **Number of distinct parameters to be estimated:** | 30 |
| **Degrees of freedom (91 - 30):** | 61 |

**Result (Default model)**

Minimum was achieved

Chi-square = 107.893

Degrees of freedom = 61

Probability level = .000

**Group number 1 (Group number 1 - Default model)**

**Estimates (Group number 1 - Default model)**

**Scalar Estimates (Group number 1 - Default model)**

**Maximum Likelihood Estimates**

**Regression Weights: (Group number 1 - Default model)**

|  |  |  | **Estimate** | **S.E.** | **C.R.** | **P** | **Label** |
| --- | --- | --- | --- | --- | --- | --- | --- |
| B | <--- | A | .642 | .072 | 8.942 | *** | par_11 |
| C | <--- | A | .402 | .105 | 3.817 | *** | par_10 |
| C | <--- | B | .629 | .130 | 4.828 | *** | par_12 |
| D | <--- | C | .561 | .070 | 8.020 | *** | par_13 |
| C1 | <--- | C | 1.000 |  |  |  |  |
| C2 | <--- | C | 1.141 | .099 | 11.546 | *** | par_1 |
| A3 | <--- | A | 1.000 |  |  |  |  |
| A2 | <--- | A | .903 | .072 | 12.559 | *** | par_2 |
| A1 | <--- | A | 1.934 | .147 | 13.177 | *** | par_3 |
| B3 | <--- | B | 1.000 |  |  |  |  |
| B2 | <--- | B | 1.591 | .144 | 11.041 | *** | par_4 |
| B1 | <--- | B | 1.195 | .107 | 11.122 | *** | par_5 |
| D1 | <--- | D | 1.000 |  |  |  |  |
| D2 | <--- | D | 1.051 | .089 | 11.783 | *** | par_6 |
| D3 | <--- | D | 1.171 | .101 | 11.546 | *** | par_7 |
| D4 | <--- | D | 1.013 | .090 | 11.310 | *** | par_8 |
| A4 | <--- | A | 1.087 | .081 | 13.458 | *** | par_9 |

**Standardized Regression Weights: (Group number 1 - Default model)**

|  |  |  | **Estimate** |
| --- | --- | --- | --- |
| B | <--- | A | .686 |
| C | <--- | A | .324 |
| C | <--- | B | .474 |
| D | <--- | C | .595 |
| C1 | <--- | C | .651 |
| C2 | <--- | C | .792 |
| A3 | <--- | A | .718 |
| A2 | <--- | A | .652 |
| A1 | <--- | A | .726 |
| B3 | <--- | B | .598 |
| B2 | <--- | B | .778 |
| B1 | <--- | B | .712 |
| D1 | <--- | D | .638 |
| D2 | <--- | D | .728 |
| D3 | <--- | D | .723 |
| D4 | <--- | D | .688 |
| A4 | <--- | A | .735 |

**Variances: (Group number 1 - Default model)**

|  |  |  | **Estimate** | **S.E.** | **C.R.** | **P** | **Label** |
| --- | --- | --- | --- | --- | --- | --- | --- |
| **A** |  |  | .106 | .013 | 8.081 | *** | par_14 |
| **e14** |  |  | .049 | .009 | 5.496 | *** | par_15 |
| **e15** |  |  | .076 | .014 | 5.498 | *** | par_16 |
| **e16** |  |  | .094 | .015 | 6.285 | *** | par_17 |
| **e8** |  |  | .222 | .019 | 11.726 | *** | par_18 |
| **e9** |  |  | .127 | .017 | 7.677 | *** | par_19 |
| **e3** |  |  | .100 | .009 | 11.597 | *** | par_20 |
| **e2** |  |  | .117 | .009 | 12.651 | *** | par_21 |
| **e1** |  |  | .356 | .031 | 11.465 | *** | par_22 |
| **e7** |  |  | .167 | .013 | 12.873 | *** | par_23 |
| **e6** |  |  | .153 | .017 | 8.934 | *** | par_24 |
| **e10** |  |  | .211 | .017 | 12.517 | *** | par_25 |
| **e11** |  |  | .143 | .013 | 10.813 | *** | par_26 |
| **e12** |  |  | .182 | .017 | 10.976 | *** | par_27 |
| **e13** |  |  | .166 | .014 | 11.679 | *** | par_28 |
| **e4** |  |  | .106 | .009 | 11.294 | *** | par_29 |
| **e5** |  |  | .129 | .012 | 10.806 | *** | par_30 |

**Squared Multiple Correlations: (Group number 1 - Default model)**

|  |  |  | **Estimate** |
| --- | --- | --- | --- |
| **B** |  |  | .471 |
| **C** |  |  | .539 |
| **D** |  |  | .354 |
| **A4** |  |  | .541 |
| **D4** |  |  | .474 |
| **D3** |  |  | .523 |
| **D2** |  |  | .529 |
| **D1** |  |  | .408 |
| **B1** |  |  | .506 |
| **B2** |  |  | .606 |
| **B3** |  |  | .357 |
| **A1** |  |  | .527 |
| **A2** |  |  | .426 |
| **A3** |  |  | .516 |
| **C2** |  |  | .627 |
| **C1** |  |  | .424 |

**Matrices (Group number 1 - Default model)**

**Residual Covariances (Group number 1 - Default model)**

|  | **A4** | **D4** | **D3** | **D2** | **D1** | **B1** | **B2** | **B3** | **A1** | **A2** | **A3** | **C2** | **C1** |
| --- | --- | --- | --- | --- | --- | --- | --- | --- | --- | --- | --- | --- | --- |
| **A4** | .000 |  |  |  |  |  |  |  |  |  |  |  |  |
| **D4** | .005 | .000 |  |  |  |  |  |  |  |  |  |  |  |
| **D3** | .001 | .009 | .000 |  |  |  |  |  |  |  |  |  |  |
| **D2** | .010 | -.008 | .002 | .000 |  |  |  |  |  |  |  |  |  |
| **D1** | .026 | .001 | -.007 | .003 | .000 |  |  |  |  |  |  |  |  |
| **B1** | -.008 | -.006 | -.012 | -.001 | .035 | .000 |  |  |  |  |  |  |  |
| **B2** | -.005 | .025 | .000 | .022 | .037 | -.004 | .000 |  |  |  |  |  |  |
| **B3** | .002 | -.026 | -.019 | -.014 | -.017 | .012 | -.004 | .000 |  |  |  |  |  |
| **A1** | .015 | -.030 | -.015 | -.002 | .061 | .024 | .003 | -.003 | .000 |  |  |  |  |
| **A2** | -.006 | -.003 | -.010 | -.006 | .009 | .004 | .005 | .002 | -.015 | .000 |  |  |  |
| **A3** | -.001 | .004 | -.012 | .004 | .020 | -.003 | .000 | -.011 | -.009 | .011 | .000 |  |  |
| **C2** | .002 | -.008 | -.020 | .003 | -.013 | -.006 | .010 | -.012 | .021 | .006 | .005 | .000 |  |
| **C1** | -.014 | .016 | .013 | .013 | .000 | -.016 | .010 | -.001 | -.035 | -.001 | -.020 | .003 | .000 |

**Standardized Residual Covariances (Group number 1 - Default model)**

|  | **A4** | **D4** | **D3** | **D2** | **D1** | **B1** | **B2** | **B3** | **A1** | **A2** | **A3** | **C2** | **C1** |
| --- | --- | --- | --- | --- | --- | --- | --- | --- | --- | --- | --- | --- | --- |
| **A4** | .000 |  |  |  |  |  |  |  |  |  |  |  |  |
| **D4** | .401 | .000 |  |  |  |  |  |  |  |  |  |  |  |
| **D3** | .068 | .504 | .000 |  |  |  |  |  |  |  |  |  |  |
| **D2** | .793 | -.515 | .112 | .000 |  |  |  |  |  |  |  |  |  |
| **D1** | 1.897 | .039 | -.356 | .181 | .000 |  |  |  |  |  |  |  |  |
| **B1** | -.675 | -.417 | -.773 | -.074 | 2.381 | .000 |  |  |  |  |  |  |  |
| **B2** | -.312 | 1.501 | -.025 | 1.335 | 2.079 | -.263 | .000 |  |  |  |  |  |  |
| **B3** | .199 | -1.915 | -1.289 | -1.073 | -1.207 | .919 | -.238 | .000 |  |  |  |  |  |
| **A1** | .686 | -1.278 | -.598 | -.086 | 2.471 | 1.104 | .094 | -.116 | .000 |  |  |  |  |
| **A2** | -.509 | -.213 | -.788 | -.546 | .741 | .340 | .334 | .213 | -.756 | .000 |  |  |  |
| **A3** | -.122 | .311 | -.890 | .312 | 1.529 | -.267 | .005 | -.931 | -.436 | 1.086 | .000 |  |  |
| **C2** | .124 | -.500 | -1.118 | .166 | -.742 | -.381 | .513 | -.810 | .833 | .430 | .383 | .000 |  |
| **C1** | -.959 | .943 | .688 | .808 | -.014 | -1.006 | .515 | -.042 | -1.307 | -.080 | -1.430 | .149 | .000 |

**Factor Score Weights (Group number 1 - Default model)**

|  | **A4** | **D4** | **D3** | **D2** | **D1** | **B1** | **B2** | **B3** | **A1** | **A2** | **A3** | **C2** | **C1** |
| --- | --- | --- | --- | --- | --- | --- | --- | --- | --- | --- | --- | --- | --- |
| **A** | .184 | .004 | .004 | .005 | .003 | .033 | .037 | .021 | .098 | .140 | .181 | .037 | .019 |
| **B** | .036 | .006 | .006 | .007 | .004 | .169 | .190 | .109 | .019 | .027 | .036 | .051 | .025 |
| **C** | .042 | .035 | .037 | .042 | .027 | .052 | .059 | .034 | .022 | .032 | .041 | .316 | .158 |
| **D** | .007 | .171 | .181 | .207 | .133 | .008 | .009 | .005 | .004 | .005 | .007 | .051 | .026 |

**Total Effects (Group number 1 - Default model)**

|  | **A** | **B** | **C** | **D** |
| --- | --- | --- | --- | --- |
| **B** | .642 | .000 | .000 | .000 |
| **C** | .806 | .629 | .000 | .000 |
| **D** | .452 | .353 | .561 | .000 |
| **A4** | 1.087 | .000 | .000 | .000 |
| **D4** | .458 | .357 | .568 | 1.013 |
| **D3** | .529 | .413 | .656 | 1.171 |
| **D2** | .475 | .371 | .589 | 1.051 |
| **D1** | .452 | .353 | .561 | 1.000 |
| **B1** | .767 | 1.195 | .000 | .000 |
| **B2** | 1.022 | 1.591 | .000 | .000 |
| **B3** | .642 | 1.000 | .000 | .000 |
| **A1** | 1.934 | .000 | .000 | .000 |
| **A2** | .903 | .000 | .000 | .000 |
| **A3** | 1.000 | .000 | .000 | .000 |
| **C2** | .920 | .718 | 1.141 | .000 |
| **C1** | .806 | .629 | 1.000 | .000 |

**Standardized Total Effects (Group number 1 - Default model)**

|  | **A** | **B** | **C** | **D** |
| --- | --- | --- | --- | --- |
| **B** | .686 | .000 | .000 | .000 |
| **C** | .648 | .474 | .000 | .000 |
| **D** | .386 | .282 | .595 | .000 |
| **A4** | .735 | .000 | .000 | .000 |
| **D4** | .266 | .194 | .410 | .688 |
| **D3** | .279 | .204 | .430 | .723 |
| **D2** | .281 | .205 | .433 | .728 |
| **D1** | .246 | .180 | .380 | .638 |
| **B1** | .488 | .712 | .000 | .000 |
| **B2** | .534 | .778 | .000 | .000 |
| **B3** | .410 | .598 | .000 | .000 |
| **A1** | .726 | .000 | .000 | .000 |
| **A2** | .652 | .000 | .000 | .000 |
| **A3** | .718 | .000 | .000 | .000 |
| **C2** | .513 | .375 | .792 | .000 |
| **C1** | .422 | .309 | .651 | .000 |

**Direct Effects (Group number 1 - Default model)**

|  | **A** | **B** | **C** | **D** |
| --- | --- | --- | --- | --- |
| **B** | .642 | .000 | .000 | .000 |
| **C** | .402 | .629 | .000 | .000 |
| **D** | .000 | .000 | .561 | .000 |
| **A4** | 1.087 | .000 | .000 | .000 |
| **D4** | .000 | .000 | .000 | 1.013 |
| **D3** | .000 | .000 | .000 | 1.171 |
| **D2** | .000 | .000 | .000 | 1.051 |
| **D1** | .000 | .000 | .000 | 1.000 |
| **B1** | .000 | 1.195 | .000 | .000 |
| **B2** | .000 | 1.591 | .000 | .000 |
| **B3** | .000 | 1.000 | .000 | .000 |
| **A1** | 1.934 | .000 | .000 | .000 |
| **A2** | .903 | .000 | .000 | .000 |
| **A3** | 1.000 | .000 | .000 | .000 |
| **C2** | .000 | .000 | 1.141 | .000 |
| **C1** | .000 | .000 | 1.000 | .000 |

**Standardized Direct Effects (Group number 1 - Default model)**

|  | **A** | **B** | **C** | **D** |
| --- | --- | --- | --- | --- |
| **B** | .686 | .000 | .000 | .000 |
| **C** | .324 | .474 | .000 | .000 |
| **D** | .000 | .000 | .595 | .000 |
| **A4** | .735 | .000 | .000 | .000 |
| **D4** | .000 | .000 | .000 | .688 |
| **D3** | .000 | .000 | .000 | .723 |
| **D2** | .000 | .000 | .000 | .728 |
| **D1** | .000 | .000 | .000 | .638 |
| **B1** | .000 | .712 | .000 | .000 |
| **B2** | .000 | .778 | .000 | .000 |
| **B3** | .000 | .598 | .000 | .000 |
| **A1** | .726 | .000 | .000 | .000 |
| **A2** | .652 | .000 | .000 | .000 |
| **A3** | .718 | .000 | .000 | .000 |
| **C2** | .000 | .000 | .792 | .000 |
| **C1** | .000 | .000 | .651 | .000 |

**Indirect Effects (Group number 1 - Default model)**

|  | **A** | **B** | **C** | **D** |
| --- | --- | --- | --- | --- |
| **B** | .000 | .000 | .000 | .000 |
| **C** | .404 | .000 | .000 | .000 |
| **D** | .452 | .353 | .000 | .000 |
| **A4** | .000 | .000 | .000 | .000 |
| **D4** | .458 | .357 | .568 | .000 |
| **D3** | .529 | .413 | .656 | .000 |
| **D2** | .475 | .371 | .589 | .000 |
| **D1** | .452 | .353 | .561 | .000 |
| **B1** | .767 | .000 | .000 | .000 |
| **B2** | 1.022 | .000 | .000 | .000 |
| **B3** | .642 | .000 | .000 | .000 |
| **A1** | .000 | .000 | .000 | .000 |
| **A2** | .000 | .000 | .000 | .000 |
| **A3** | .000 | .000 | .000 | .000 |
| **C2** | .920 | .718 | .000 | .000 |
| **C1** | .806 | .629 | .000 | .000 |

**Standardized Indirect Effects (Group number 1 - Default model)**

|  | **A** | **B** | **C** | **D** |
| --- | --- | --- | --- | --- |
| **B** | .000 | .000 | .000 | .000 |
| **C** | .325 | .000 | .000 | .000 |
| **D** | .386 | .282 | .000 | .000 |
| **A4** | .000 | .000 | .000 | .000 |
| **D4** | .266 | .194 | .410 | .000 |
| **D3** | .279 | .204 | .430 | .000 |
| **D2** | .281 | .205 | .433 | .000 |
| **D1** | .246 | .180 | .380 | .000 |
| **B1** | .488 | .000 | .000 | .000 |
| **B2** | .534 | .000 | .000 | .000 |
| **B3** | .410 | .000 | .000 | .000 |
| **A1** | .000 | .000 | .000 | .000 |
| **A2** | .000 | .000 | .000 | .000 |
| **A3** | .000 | .000 | .000 | .000 |
| **C2** | .513 | .375 | .000 | .000 |
| **C1** | .422 | .309 | .000 | .000 |

**Modification Indices (Group number 1 - Default model)**

**Covariances: (Group number 1 - Default model)**

|  |  |  | **M.I.** | **Par Change** |
| --- | --- | --- | --- | --- |
| e10 | <--> | A | 7.175 | .022 |
| e10 | <--> | e15 | 7.858 | -.025 |
| e5 | <--> | e10 | 9.410 | .029 |
| e6 | <--> | e15 | 4.830 | .018 |
| e6 | <--> | e16 | 4.628 | .018 |
| e6 | <--> | e13 | 4.435 | .021 |
| e7 | <--> | e16 | 6.821 | -.020 |
| e7 | <--> | e10 | 4.035 | -.020 |
| e7 | <--> | e5 | 4.288 | .017 |
| e1 | <--> | e4 | 4.060 | .022 |
| e1 | <--> | e13 | 6.900 | -.037 |
| e1 | <--> | e10 | 7.708 | .043 |
| e1 | <--> | e5 | 5.118 | .028 |
| e3 | <--> | e2 | 7.005 | .016 |

**Variances: (Group number 1 - Default model)**

|  |  |  | **M.I.** | **Par Change** |
| --- | --- | --- | --- | --- |

**Regression Weights: (Group number 1 - Default model)**

|  |  |  | **M.I.** | **Par Change** |
| --- | --- | --- | --- | --- |
| D1 | <--- | A | 7.175 | .209 |
| D1 | <--- | B | 4.753 | .185 |
| D1 | <--- | A4 | 4.179 | .098 |
| D1 | <--- | B1 | 10.996 | .150 |
| D1 | <--- | A1 | 12.186 | .093 |
| B1 | <--- | D1 | 4.323 | .066 |
| B2 | <--- | D | 5.307 | .147 |
| B2 | <--- | D4 | 7.915 | .110 |
| B2 | <--- | D2 | 4.053 | .080 |
| B3 | <--- | D | 6.107 | -.146 |
| B3 | <--- | D4 | 6.927 | -.095 |
| B3 | <--- | D1 | 8.163 | -.097 |
| C1 | <--- | A1 | 4.384 | -.058 |

**Minimization History (Default model)**

| **Iteration** |  | **Negative eigenvalues** | **Condition #** | **Smallest eigenvalue** | **Diameter** | **F** | **NTries** | **Ratio** |
| --- | --- | --- | --- | --- | --- | --- | --- | --- |
| **0** | e | 8 |  | -.362 | 9999.000 | 2148.923 | 0 | 9999.000 |
| **1** | e | 3 |  | -.039 | 2.403 | 786.256 | 20 | .531 |
| **2** | e | 0 | 66.356 |  | 1.463 | 284.540 | 5 | .708 |
| **3** | e | 0 | 50.171 |  | .556 | 204.465 | 3 | .000 |
| **4** | e | 0 | 38.632 |  | .584 | 120.990 | 1 | 1.077 |
| **5** | e | 0 | 43.123 |  | .260 | 108.481 | 1 | 1.095 |
| **6** | e | 0 | 58.965 |  | .094 | 107.900 | 1 | 1.053 |
| **7** | e | 0 | 58.228 |  | .011 | 107.893 | 1 | 1.009 |
| **8** | e | 0 | 60.160 |  | .000 | 107.893 | 1 | 1.000 |

**Pairwise Parameter Comparisons (Default model)**

**Variance-covariance Matrix of Estimates (Default model)**

|  | **par_1** | **par_2** | **par_3** | **par_4** | **par_5** | **par_6** | **par_7** | **par_8** | **par_9** | **par_10** | **par_11** | **par_12** | **par_13** | **par_14** | **par_15** | **par_16** | **par_17** | **par_18** | **par_19** | **par_20** | **par_21** | **par_22** | **par_23** | **par_24** | **par_25** | **par_26** | **par_27** | **par_28** | **par_29** | **par_30** |
| --- | --- | --- | --- | --- | --- | --- | --- | --- | --- | --- | --- | --- | --- | --- | --- | --- | --- | --- | --- | --- | --- | --- | --- | --- | --- | --- | --- | --- | --- | --- |
| **par_1** | .010 |  |  |  |  |  |  |  |  |  |  |  |  |  |  |  |  |  |  |  |  |  |  |  |  |  |  |  |  |  |
| **par_2** | .000 | .005 |  |  |  |  |  |  |  |  |  |  |  |  |  |  |  |  |  |  |  |  |  |  |  |  |  |  |  |  |
| **par_3** | .000 | .005 | .022 |  |  |  |  |  |  |  |  |  |  |  |  |  |  |  |  |  |  |  |  |  |  |  |  |  |  |  |
| **par_4** | .000 | .000 | .000 | .021 |  |  |  |  |  |  |  |  |  |  |  |  |  |  |  |  |  |  |  |  |  |  |  |  |  |  |
| **par_5** | .000 | .000 | .000 | .010 | .012 |  |  |  |  |  |  |  |  |  |  |  |  |  |  |  |  |  |  |  |  |  |  |  |  |  |
| **par_6** | .000 | .000 | .000 | .000 | .000 | .008 |  |  |  |  |  |  |  |  |  |  |  |  |  |  |  |  |  |  |  |  |  |  |  |  |
| **par_7** | .000 | .000 | .000 | .000 | .000 | .005 | .010 |  |  |  |  |  |  |  |  |  |  |  |  |  |  |  |  |  |  |  |  |  |  |  |
| **par_8** | .000 | .000 | .000 | .000 | .000 | .004 | .005 | .008 |  |  |  |  |  |  |  |  |  |  |  |  |  |  |  |  |  |  |  |  |  |  |
| **par_9** | .000 | .003 | .007 | .000 | .000 | .000 | .000 | .000 | .007 |  |  |  |  |  |  |  |  |  |  |  |  |  |  |  |  |  |  |  |  |  |
| **par_10** | -.001 | .001 | .002 | -.001 | .000 | .000 | .000 | .000 | .001 | .011 |  |  |  |  |  |  |  |  |  |  |  |  |  |  |  |  |  |  |  |  |
| **par_11** | .000 | .002 | .004 | -.006 | -.004 | .000 | .000 | .000 | .002 | .001 | .005 |  |  |  |  |  |  |  |  |  |  |  |  |  |  |  |  |  |  |  |
| **par_12** | -.004 | .000 | .000 | .007 | .004 | .000 | .000 | .000 | .000 | -.009 | -.002 | .017 |  |  |  |  |  |  |  |  |  |  |  |  |  |  |  |  |  |  |
| **par_13** | .002 | .000 | .000 | .000 | .000 | -.003 | -.003 | -.003 | .000 | -.001 | .000 | -.001 | .005 |  |  |  |  |  |  |  |  |  |  |  |  |  |  |  |  |  |
| **par_14** | .000 | -.001 | -.001 | .000 | .000 | .000 | .000 | .000 | -.001 | .000 | .000 | .000 | .000 | .000 |  |  |  |  |  |  |  |  |  |  |  |  |  |  |  |  |
| **par_15** | .000 | .000 | .000 | -.001 | -.001 | .000 | .000 | .000 | .000 | .000 | .000 | .000 | .000 | .000 | .000 |  |  |  |  |  |  |  |  |  |  |  |  |  |  |  |
| **par_16** | -.001 | .000 | .000 | .000 | .000 | .000 | .000 | .000 | .000 | .000 | .000 | .000 | .000 | .000 | .000 | .000 |  |  |  |  |  |  |  |  |  |  |  |  |  |  |
| **par_17** | .000 | .000 | .000 | .000 | .000 | -.001 | -.001 | -.001 | .000 | .000 | .000 | .000 | .000 | .000 | .000 | .000 | .000 |  |  |  |  |  |  |  |  |  |  |  |  |  |
| **par_18** | .001 | .000 | .000 | .000 | .000 | .000 | .000 | .000 | .000 | .000 | .000 | .000 | .000 | .000 | .000 | .000 | .000 | .000 |  |  |  |  |  |  |  |  |  |  |  |  |
| **par_19** | -.001 | .000 | .000 | .000 | .000 | .000 | .000 | .000 | .000 | .000 | .000 | .000 | .000 | .000 | .000 | .000 | .000 | .000 | .000 |  |  |  |  |  |  |  |  |  |  |  |
| **par_20** | .000 | .000 | .000 | .000 | .000 | .000 | .000 | .000 | .000 | .000 | .000 | .000 | .000 | .000 | .000 | .000 | .000 | .000 | .000 | .000 |  |  |  |  |  |  |  |  |  |  |
| **par_21** | .000 | .000 | .000 | .000 | .000 | .000 | .000 | .000 | .000 | .000 | .000 | .000 | .000 | .000 | .000 | .000 | .000 | .000 | .000 | .000 | .000 |  |  |  |  |  |  |  |  |  |
| **par_22** | .000 | .000 | -.001 | .000 | .000 | .000 | .000 | .000 | .000 | .000 | .000 | .000 | .000 | .000 | .000 | .000 | .000 | .000 | .000 | .000 | .000 | .001 |  |  |  |  |  |  |  |  |
| **par_23** | .000 | .000 | .000 | .000 | .000 | .000 | .000 | .000 | .000 | .000 | .000 | .000 | .000 | .000 | .000 | .000 | .000 | .000 | .000 | .000 | .000 | .000 | .000 |  |  |  |  |  |  |  |
| **par_24** | .000 | .000 | .000 | -.001 | .000 | .000 | .000 | .000 | .000 | .000 | .000 | .000 | .000 | .000 | .000 | .000 | .000 | .000 | .000 | .000 | .000 | .000 | .000 | .000 |  |  |  |  |  |  |
| **par_25** | .000 | .000 | .000 | .000 | .000 | .000 | .000 | .000 | .000 | .000 | .000 | .000 | .000 | .000 | .000 | .000 | .000 | .000 | .000 | .000 | .000 | .000 | .000 | .000 | .000 |  |  |  |  |  |
| **par_26** | .000 | .000 | .000 | .000 | .000 | .000 | .000 | .000 | .000 | .000 | .000 | .000 | .000 | .000 | .000 | .000 | .000 | .000 | .000 | .000 | .000 | .000 | .000 | .000 | .000 | .000 |  |  |  |  |
| **par_27** | .000 | .000 | .000 | .000 | .000 | .000 | .000 | .000 | .000 | .000 | .000 | .000 | .000 | .000 | .000 | .000 | .000 | .000 | .000 | .000 | .000 | .000 | .000 | .000 | .000 | .000 | .000 |  |  |  |
| **par_28** | .000 | .000 | .000 | .000 | .000 | .000 | .000 | .000 | .000 | .000 | .000 | .000 | .000 | .000 | .000 | .000 | .000 | .000 | .000 | .000 | .000 | .000 | .000 | .000 | .000 | .000 | .000 | .000 |  |  |
| **par_29** | .000 | .000 | .000 | .000 | .000 | .000 | .000 | .000 | .000 | .000 | .000 | .000 | .000 | .000 | .000 | .000 | .000 | .000 | .000 | .000 | .000 | .000 | .000 | .000 | .000 | .000 | .000 | .000 | .000 |  |
| **par_30** | .000 | .000 | .000 | .000 | .000 | .000 | .000 | .000 | .000 | .000 | .000 | .000 | .000 | .000 | .000 | .000 | .000 | .000 | .000 | .000 | .000 | .000 | .000 | .000 | .000 | .000 | .000 | .000 | .000 | .000 |

**Critical Ratios for Differences between Parameters (Default model)**

|  | **par_1** | **par_2** | **par_3** | **par_4** | **par_5** | **par_6** | **par_7** | **par_8** | **par_9** | **par_10** | **par_11** | **par_12** | **par_13** | **par_14** | **par_15** | **par_16** | **par_17** | **par_18** | **par_19** | **par_20** | **par_21** | **par_22** | **par_23** | **par_24** | **par_25** | **par_26** | **par_27** | **par_28** | **par_29** | **par_30** |
| --- | --- | --- | --- | --- | --- | --- | --- | --- | --- | --- | --- | --- | --- | --- | --- | --- | --- | --- | --- | --- | --- | --- | --- | --- | --- | --- | --- | --- | --- | --- |
| **par_1** | .000 |  |  |  |  |  |  |  |  |  |  |  |  |  |  |  |  |  |  |  |  |  |  |  |  |  |  |  |  |  |
| **par_2** | -1.937 | .000 |  |  |  |  |  |  |  |  |  |  |  |  |  |  |  |  |  |  |  |  |  |  |  |  |  |  |  |  |
| **par_3** | 4.493 | 7.768 | .000 |  |  |  |  |  |  |  |  |  |  |  |  |  |  |  |  |  |  |  |  |  |  |  |  |  |  |  |
| **par_4** | 2.571 | 4.263 | -1.655 | .000 |  |  |  |  |  |  |  |  |  |  |  |  |  |  |  |  |  |  |  |  |  |  |  |  |  |  |
| **par_5** | .374 | 2.254 | -4.067 | -3.445 | .000 |  |  |  |  |  |  |  |  |  |  |  |  |  |  |  |  |  |  |  |  |  |  |  |  |  |
| **par_6** | -.672 | 1.293 | -5.134 | -3.183 | -1.026 | .000 |  |  |  |  |  |  |  |  |  |  |  |  |  |  |  |  |  |  |  |  |  |  |  |  |
| **par_7** | .212 | 2.151 | -4.277 | -2.383 | -.165 | 1.378 | .000 |  |  |  |  |  |  |  |  |  |  |  |  |  |  |  |  |  |  |  |  |  |  |  |
| **par_8** | -.953 | .959 | -5.347 | -3.405 | -1.296 | -.450 | -1.810 | .000 |  |  |  |  |  |  |  |  |  |  |  |  |  |  |  |  |  |  |  |  |  |  |
| **par_9** | -.420 | 2.258 | -6.908 | -3.042 | -.802 | .294 | -.646 | .608 | .000 |  |  |  |  |  |  |  |  |  |  |  |  |  |  |  |  |  |  |  |  |  |
| **par_10** | -4.827 | -4.200 | -9.160 | -6.521 | -5.255 | -4.689 | -5.243 | -4.405 | -5.576 | .000 |  |  |  |  |  |  |  |  |  |  |  |  |  |  |  |  |  |  |  |  |
| **par_11** | -4.084 | -3.099 | -9.359 | -4.898 | -3.534 | -3.578 | -4.261 | -3.236 | -5.099 | 1.964 | .000 |  |  |  |  |  |  |  |  |  |  |  |  |  |  |  |  |  |  |  |
| **par_12** | -2.711 | -1.835 | -6.614 | -6.167 | -3.914 | -2.668 | -3.272 | -2.429 | -2.982 | 1.062 | -.077 | .000 |  |  |  |  |  |  |  |  |  |  |  |  |  |  |  |  |  |  |
| **par_13** | -5.861 | -3.410 | -8.447 | -6.475 | -4.976 | -3.658 | -4.166 | -3.371 | -4.932 | 1.199 | -.808 | -.438 | .000 |  |  |  |  |  |  |  |  |  |  |  |  |  |  |  |  |  |
| **par_14** | -10.382 | -10.000 | -11.768 | -10.270 | -10.062 | -10.482 | -10.412 | -10.020 | -10.948 | -2.727 | -6.853 | -4.001 | -6.391 | .000 |  |  |  |  |  |  |  |  |  |  |  |  |  |  |  |  |
| **par_15** | -11.003 | -11.768 | -12.809 | -10.262 | -10.114 | -11.178 | -11.021 | -10.709 | -12.783 | -3.369 | -8.545 | -4.335 | -7.249 | -3.614 | .000 |  |  |  |  |  |  |  |  |  |  |  |  |  |  |  |
| **par_16** | -9.923 | -11.309 | -12.607 | -10.431 | -10.325 | -10.836 | -10.736 | -10.375 | -12.345 | -3.127 | -7.781 | -4.267 | -6.332 | -1.607 | 1.634 | .000 |  |  |  |  |  |  |  |  |  |  |  |  |  |  |
| **par_17** | -10.582 | -11.020 | -12.475 | -10.323 | -10.147 | -9.626 | -9.668 | -9.247 | -12.083 | -2.901 | -7.480 | -4.070 | -6.902 | -.609 | 2.577 | .895 | .000 |  |  |  |  |  |  |  |  |  |  |  |  |  |
| **par_18** | -9.769 | -9.141 | -11.569 | -9.419 | -8.930 | -9.081 | -9.176 | -8.620 | -10.413 | -1.680 | -5.644 | -3.028 | -4.871 | 5.046 | 8.247 | 5.501 | 5.398 | .000 |  |  |  |  |  |  |  |  |  |  |  |  |
| **par_19** | -9.453 | -10.527 | -12.226 | -10.117 | -9.820 | -10.150 | -10.131 | -9.706 | -11.663 | -2.573 | -6.981 | -3.914 | -6.135 | .986 | 4.126 | 2.364 | 1.405 | -3.504 | .000 |  |  |  |  |  |  |  |  |  |  |  |
| **par_20** | -10.495 | -11.335 | -12.690 | -10.324 | -10.160 | -10.617 | -10.525 | -10.150 | -12.522 | -2.884 | -7.669 | -4.048 | -6.544 | -.369 | 4.015 | 1.482 | .329 | -5.891 | -1.465 | .000 |  |  |  |  |  |  |  |  |  |  |
| **par_21** | -10.324 | -10.612 | -12.420 | -10.210 | -9.999 | -10.419 | -10.350 | -9.952 | -12.035 | -2.703 | -7.274 | -3.920 | -6.299 | .656 | 5.249 | 2.479 | 1.297 | -5.021 | -.535 | 1.391 | .000 |  |  |  |  |  |  |  |  |  |
| **par_22** | -7.549 | -7.003 | -9.953 | -8.391 | -7.483 | -7.361 | -7.682 | -6.936 | -8.243 | -.420 | -3.599 | -2.034 | -2.671 | 7.649 | 9.481 | 8.245 | 7.591 | 3.661 | 6.553 | 7.707 | 7.175 | .000 |  |  |  |  |  |  |  |  |
| **par_23** | -9.770 | -10.068 | -11.988 | -10.081 | -9.717 | -9.806 | -9.814 | -9.347 | -11.231 | -2.199 | -6.343 | -3.586 | -5.550 | 3.307 | 6.776 | 4.744 | 3.677 | -2.418 | 1.916 | 4.313 | 3.153 | -5.620 | .000 |  |  |  |  |  |  |  |
| **par_24** | -9.881 | -10.163 | -12.076 | -9.519 | -9.624 | -9.890 | -9.902 | -9.430 | -11.328 | -2.331 | -6.848 | -3.596 | -5.649 | 2.161 | 5.450 | 3.561 | 2.609 | -2.730 | 1.077 | 2.798 | 1.860 | -5.688 | -.619 | .000 |  |  |  |  |  |  |
| **par_25** | -9.276 | -9.365 | -11.658 | -9.507 | -9.039 | -9.671 | -9.742 | -9.184 | -10.610 | -1.787 | -5.839 | -3.178 | -4.696 | 4.924 | 8.496 | 6.311 | 4.703 | -.431 | 3.539 | 5.897 | 4.915 | -4.096 | 2.083 | 2.434 | .000 |  |  |  |  |  |
| **par_26** | -9.997 | -10.398 | -12.153 | -10.007 | -9.718 | -9.707 | -10.082 | -9.687 | -11.533 | -2.442 | -6.835 | -3.713 | -5.891 | 1.980 | 5.876 | 3.532 | 2.488 | -3.427 | .764 | 2.749 | 1.625 | -6.314 | -1.296 | -.459 | -3.130 | .000 |  |  |  |  |
| **par_27** | -9.573 | -9.771 | -11.860 | -9.721 | -9.322 | -9.561 | -9.210 | -9.045 | -10.975 | -2.066 | -6.239 | -3.407 | -5.392 | 3.588 | 7.040 | 4.899 | 3.986 | -1.614 | 2.360 | 4.405 | 3.435 | -4.950 | .711 | 1.214 | -1.192 | 1.747 | .000 |  |  |  |
| **par_28** | -9.768 | -10.054 | -11.991 | -9.838 | -9.493 | -9.866 | -9.786 | -9.005 | -11.229 | -2.221 | -6.503 | -3.531 | -5.574 | 3.101 | 6.964 | 4.562 | 3.523 | -2.379 | 1.799 | 4.006 | 2.914 | -5.554 | -.042 | .597 | -2.008 | 1.112 | -.714 | .000 |  |  |
| **par_29** | -10.430 | -11.006 | -12.381 | -10.285 | -10.103 | -10.532 | -10.451 | -10.067 | -11.691 | -2.800 | -7.393 | -3.994 | -6.436 | .018 | 4.305 | 1.844 | .704 | -5.487 | -1.073 | .504 | -.753 | -7.709 | -3.795 | -2.384 | -5.431 | -2.256 | -3.963 | -3.504 | .000 |  |
| **par_30** | -10.141 | -10.612 | -12.243 | -10.230 | -9.643 | -10.246 | -10.201 | -9.784 | -11.732 | -2.548 | -7.033 | -3.865 | -6.081 | 1.306 | 5.201 | 2.879 | 1.836 | -4.131 | .115 | 2.003 | .824 | -6.858 | -2.196 | -1.018 | -3.975 | -.771 | -2.580 | -1.985 | 1.494 | .000 |

**Model Fit Summary**

**CMIN**

| **Model** | **NPAR** | **CMIN** | **DF** | **P** | **CMIN/DF** |
| --- | --- | --- | --- | --- | --- |
| **Default model** | 30 | 107.893 | 61 | .000 | 1.769 |
| **Saturated model** | 91 | .000 | 0 |  |  |
| **Independence model** | 13 | 2028.542 | 78 | .000 | 26.007 |

**RMR, GFI**

| **Model** | **RMR** | **GFI** | **AGFI** | **PGFI** |
| --- | --- | --- | --- | --- |
| **Default model** | .014 | .965 | .948 | .647 |
| **Saturated model** | .000 | 1.000 |  |  |
| **Independence model** | .105 | .423 | .327 | .363 |

**Baseline Comparisons**

| **Model** | **NFI Delta1** | **RFI rho1** | **IFI Delta2** | **TLI rho2** | **CFI** |
| --- | --- | --- | --- | --- | --- |
| **Default model** | .947 | .932 | .976 | .969 | .976 |
| **Saturated model** | 1.000 |  | 1.000 |  | 1.000 |
| **Independence model** | .000 | .000 | .000 | .000 | .000 |

**Parsimony-Adjusted Measures**

| **Model** | **PRATIO** | **PNFI** | **PCFI** |
| --- | --- | --- | --- |
| **Default model** | .782 | .740 | .763 |
| **Saturated model** | .000 | .000 | .000 |
| **Independence model** | 1.000 | .000 | .000 |

**NCP**

| **Model** | **NCP** | **LO 90** | **HI 90** |
| --- | --- | --- | --- |
| **Default model** | 46.893 | 21.805 | 79.832 |
| **Saturated model** | .000 | .000 | .000 |
| **Independence model** | 1950.542 | 1807.436 | 2101.007 |

**FMIN**

| **Model** | **FMIN** | **F0** | **LO 90** | **HI 90** |
| --- | --- | --- | --- | --- |
| **Default model** | .237 | .103 | .048 | .175 |
| **Saturated model** | .000 | .000 | .000 | .000 |
| **Independence model** | 4.449 | 4.278 | 3.964 | 4.607 |

**RMSEA**

| **Model** | **RMSEA** | **LO 90** | **HI 90** | **PCLOSE** |
| --- | --- | --- | --- | --- |
| **Default model** | .041 | .028 | .054 | .875 |
| **Independence model** | .234 | .225 | .243 | .000 |

**AIC**

| **Model** | **AIC** | **BCC** | **BIC** | **CAIC** |
| --- | --- | --- | --- | --- |
| **Default model** | 167.893 | 169.793 | 291.634 | 321.634 |
| **Saturated model** | 182.000 | 187.765 | 557.346 | 648.346 |
| **Independence model** | 2054.542 | 2055.365 | 2108.163 | 2121.163 |

**ECVI**

| **Model** | **ECVI** | **LO 90** | **HI 90** | **MECVI** |
| --- | --- | --- | --- | --- |
| **Default model** | .368 | .313 | .440 | .372 |
| **Saturated model** | .399 | .399 | .399 | .412 |
| **Independence model** | 4.506 | 4.192 | 4.836 | 4.507 |

**HOELTER**

| **Model** | **HOELTER .05** | **HOELTER .01** |
| --- | --- | --- |
| **Default model** | 340 | 379 |
| **Independence model** | 23 | 25 |

**Execution time summary**

| **Minimization:** | .021 |
| --- | --- |
| **Miscellaneous:** | .229 |
| **Bootstrap:** | .000 |
| **Total:** | .250 |
