# Supplementary material for: Self-health promotion: A study on the mode of acquiring sports health knowledge and skills among older adults members of sports communities
Source: PLoS One. 2024 Jul 11;19(7):e0304814. doi: 10.1371/journal.pone.0304814 (PMC11239043; doi:10.1371/journal.pone.0304814)
Supplement: S1 File — (ZIP) [file pone.0304814.s001.zip › data-Self-health Promotion/fuhaoqsn.AmosOutput]

fuhaoqsn.amw


# C:\Users\Administrator\Desktop\健康促进\fuhaoqsn.amw

## Analysis Summary

## Date and Time

Date: 2024年5月2日

Time: 7:12:24

## Title

fuhaoqsn: 2024年5月2日 7:12

## Groups

## Group number 1 (Group number 1)

## Notes for Group (Group number 1)

The model is recursive.

Sample size = 457

## Variable Summary (Group number 1)

## Your model contains the following variables (Group number 1)

Observed, endogenous variables

C1

C2

A3

A2

A1

B3

B2

B1

D1

D2

D3

D4

A4

Unobserved, endogenous variables

C

B

D

Unobserved, exogenous variables

e8

e9

A

e3

e2

e1

e7

e6

e10

e11

e12

e13

e4

e14

e15

e16

e5

## Variable counts (Group number 1)

|  |  |
| --- | --- |
| Number of variables in your model: | 33 |
| Number of observed variables: | 13 |
| Number of unobserved variables: | 20 |
| Number of exogenous variables: | 17 |
| Number of endogenous variables: | 16 |

## Parameter Summary (Group number 1)

|  | Weights | Covariances | Variances | Means | Intercepts | Total |
| --- | --- | --- | --- | --- | --- | --- |
| Fixed | 20 | 0 | 0 | 0 | 0 | 20 |
| Labeled | 0 | 0 | 0 | 0 | 0 | 0 |
| Unlabeled | 13 | 0 | 17 | 0 | 0 | 30 |
| Total | 33 | 0 | 17 | 0 | 0 | 50 |

## Models

## Default model (Default model)

## Notes for Model (Default model)

## Computation of degrees of freedom (Default model)

|  |  |
| --- | --- |
| Number of distinct sample moments: | 91 |
| Number of distinct parameters to be estimated: | 30 |
| Degrees of freedom (91 - 30): | 61 |

## Result (Default model)

Minimum was achieved

Chi-square = 107.893

Degrees of freedom = 61

Probability level = .000

## Group number 1 (Group number 1 - Default model)

## Estimates (Group number 1 - Default model)

## Scalar Estimates (Group number 1 - Default model)

## Maximum Likelihood Estimates

## Regression Weights: (Group number 1 - Default model)

|  |  |  | Estimate | S.E. | C.R. | P | Label |
| --- | --- | --- | --- | --- | --- | --- | --- |
| B | <--- | A | .642 | .072 | 8.942 | \*\*\* | par\_11 |
| C | <--- | A | .402 | .105 | 3.817 | \*\*\* | par\_10 |
| C | <--- | B | .629 | .130 | 4.828 | \*\*\* | par\_12 |
| D | <--- | C | .561 | .070 | 8.020 | \*\*\* | par\_13 |
| C1 | <--- | C | 1.000 |  |
| C2 | <--- | C | 1.141 | .099 | 11.546 | \*\*\* | par\_1 |
| A3 | <--- | A | 1.000 |  |
| A2 | <--- | A | .903 | .072 | 12.559 | \*\*\* | par\_2 |
| A1 | <--- | A | 1.934 | .147 | 13.177 | \*\*\* | par\_3 |
| B3 | <--- | B | 1.000 |  |
| B2 | <--- | B | 1.591 | .144 | 11.041 | \*\*\* | par\_4 |
| B1 | <--- | B | 1.195 | .107 | 11.122 | \*\*\* | par\_5 |
| D1 | <--- | D | 1.000 |  |
| D2 | <--- | D | 1.051 | .089 | 11.783 | \*\*\* | par\_6 |
| D3 | <--- | D | 1.171 | .101 | 11.546 | \*\*\* | par\_7 |
| D4 | <--- | D | 1.013 | .090 | 11.310 | \*\*\* | par\_8 |
| A4 | <--- | A | 1.087 | .081 | 13.458 | \*\*\* | par\_9 |

## Standardized Regression Weights: (Group number 1 - Default model)

|  |  |  | Estimate |
| --- | --- | --- | --- |
| B | <--- | A | .686 |
| C | <--- | A | .324 |
| C | <--- | B | .474 |
| D | <--- | C | .595 |
| C1 | <--- | C | .651 |
| C2 | <--- | C | .792 |
| A3 | <--- | A | .718 |
| A2 | <--- | A | .652 |
| A1 | <--- | A | .726 |
| B3 | <--- | B | .598 |
| B2 | <--- | B | .778 |
| B1 | <--- | B | .712 |
| D1 | <--- | D | .638 |
| D2 | <--- | D | .728 |
| D3 | <--- | D | .723 |
| D4 | <--- | D | .688 |
| A4 | <--- | A | .735 |

## Variances: (Group number 1 - Default model)

|  |  |  | Estimate | S.E. | C.R. | P | Label |
| --- | --- | --- | --- | --- | --- | --- | --- |
| A |  |  | .106 | .013 | 8.081 | \*\*\* | par\_14 |
| e14 |  |  | .049 | .009 | 5.496 | \*\*\* | par\_15 |
| e15 |  |  | .076 | .014 | 5.498 | \*\*\* | par\_16 |
| e16 |  |  | .094 | .015 | 6.285 | \*\*\* | par\_17 |
| e8 |  |  | .222 | .019 | 11.726 | \*\*\* | par\_18 |
| e9 |  |  | .127 | .017 | 7.677 | \*\*\* | par\_19 |
| e3 |  |  | .100 | .009 | 11.597 | \*\*\* | par\_20 |
| e2 |  |  | .117 | .009 | 12.651 | \*\*\* | par\_21 |
| e1 |  |  | .356 | .031 | 11.465 | \*\*\* | par\_22 |
| e7 |  |  | .167 | .013 | 12.873 | \*\*\* | par\_23 |
| e6 |  |  | .153 | .017 | 8.934 | \*\*\* | par\_24 |
| e10 |  |  | .211 | .017 | 12.517 | \*\*\* | par\_25 |
| e11 |  |  | .143 | .013 | 10.813 | \*\*\* | par\_26 |
| e12 |  |  | .182 | .017 | 10.976 | \*\*\* | par\_27 |
| e13 |  |  | .166 | .014 | 11.679 | \*\*\* | par\_28 |
| e4 |  |  | .106 | .009 | 11.294 | \*\*\* | par\_29 |
| e5 |  |  | .129 | .012 | 10.806 | \*\*\* | par\_30 |

## Squared Multiple Correlations: (Group number 1 - Default model)

|  |  |  | Estimate |
| --- | --- | --- | --- |
| B |  |  | .471 |
| C |  |  | .539 |
| D |  |  | .354 |
| A4 |  |  | .541 |
| D4 |  |  | .474 |
| D3 |  |  | .523 |
| D2 |  |  | .529 |
| D1 |  |  | .408 |
| B1 |  |  | .506 |
| B2 |  |  | .606 |
| B3 |  |  | .357 |
| A1 |  |  | .527 |
| A2 |  |  | .426 |
| A3 |  |  | .516 |
| C2 |  |  | .627 |
| C1 |  |  | .424 |

## Matrices (Group number 1 - Default model)

## Residual Covariances (Group number 1 - Default model)

|  | A4 | D4 | D3 | D2 | D1 | B1 | B2 | B3 | A1 | A2 | A3 | C2 | C1 |
| --- | --- | --- | --- | --- | --- | --- | --- | --- | --- | --- | --- | --- | --- |
| A4 | .000 |
| D4 | .005 | .000 |
| D3 | .001 | .009 | .000 |
| D2 | .010 | -.008 | .002 | .000 |
| D1 | .026 | .001 | -.007 | .003 | .000 |
| B1 | -.008 | -.006 | -.012 | -.001 | .035 | .000 |
| B2 | -.005 | .025 | .000 | .022 | .037 | -.004 | .000 |
| B3 | .002 | -.026 | -.019 | -.014 | -.017 | .012 | -.004 | .000 |
| A1 | .015 | -.030 | -.015 | -.002 | .061 | .024 | .003 | -.003 | .000 |
| A2 | -.006 | -.003 | -.010 | -.006 | .009 | .004 | .005 | .002 | -.015 | .000 |
| A3 | -.001 | .004 | -.012 | .004 | .020 | -.003 | .000 | -.011 | -.009 | .011 | .000 |
| C2 | .002 | -.008 | -.020 | .003 | -.013 | -.006 | .010 | -.012 | .021 | .006 | .005 | .000 |
| C1 | -.014 | .016 | .013 | .013 | .000 | -.016 | .010 | -.001 | -.035 | -.001 | -.020 | .003 | .000 |

## Standardized Residual Covariances (Group number 1 - Default model)

|  | A4 | D4 | D3 | D2 | D1 | B1 | B2 | B3 | A1 | A2 | A3 | C2 | C1 |
| --- | --- | --- | --- | --- | --- | --- | --- | --- | --- | --- | --- | --- | --- |
| A4 | .000 |
| D4 | .401 | .000 |
| D3 | .068 | .504 | .000 |
| D2 | .793 | -.515 | .112 | .000 |
| D1 | 1.897 | .039 | -.356 | .181 | .000 |
| B1 | -.675 | -.417 | -.773 | -.074 | 2.381 | .000 |
| B2 | -.312 | 1.501 | -.025 | 1.335 | 2.079 | -.263 | .000 |
| B3 | .199 | -1.915 | -1.289 | -1.073 | -1.207 | .919 | -.238 | .000 |
| A1 | .686 | -1.278 | -.598 | -.086 | 2.471 | 1.104 | .094 | -.116 | .000 |
| A2 | -.509 | -.213 | -.788 | -.546 | .741 | .340 | .334 | .213 | -.756 | .000 |
| A3 | -.122 | .311 | -.890 | .312 | 1.529 | -.267 | .005 | -.931 | -.436 | 1.086 | .000 |
| C2 | .124 | -.500 | -1.118 | .166 | -.742 | -.381 | .513 | -.810 | .833 | .430 | .383 | .000 |
| C1 | -.959 | .943 | .688 | .808 | -.014 | -1.006 | .515 | -.042 | -1.307 | -.080 | -1.430 | .149 | .000 |

## Factor Score Weights (Group number 1 - Default model)

|  | A4 | D4 | D3 | D2 | D1 | B1 | B2 | B3 | A1 | A2 | A3 | C2 | C1 |
| --- | --- | --- | --- | --- | --- | --- | --- | --- | --- | --- | --- | --- | --- |
| A | .184 | .004 | .004 | .005 | .003 | .033 | .037 | .021 | .098 | .140 | .181 | .037 | .019 |
| B | .036 | .006 | .006 | .007 | .004 | .169 | .190 | .109 | .019 | .027 | .036 | .051 | .025 |
| C | .042 | .035 | .037 | .042 | .027 | .052 | .059 | .034 | .022 | .032 | .041 | .316 | .158 |
| D | .007 | .171 | .181 | .207 | .133 | .008 | .009 | .005 | .004 | .005 | .007 | .051 | .026 |

## Total Effects (Group number 1 - Default model)

|  | A | B | C | D |
| --- | --- | --- | --- | --- |
| B | .642 | .000 | .000 | .000 |
| C | .806 | .629 | .000 | .000 |
| D | .452 | .353 | .561 | .000 |
| A4 | 1.087 | .000 | .000 | .000 |
| D4 | .458 | .357 | .568 | 1.013 |
| D3 | .529 | .413 | .656 | 1.171 |
| D2 | .475 | .371 | .589 | 1.051 |
| D1 | .452 | .353 | .561 | 1.000 |
| B1 | .767 | 1.195 | .000 | .000 |
| B2 | 1.022 | 1.591 | .000 | .000 |
| B3 | .642 | 1.000 | .000 | .000 |
| A1 | 1.934 | .000 | .000 | .000 |
| A2 | .903 | .000 | .000 | .000 |
| A3 | 1.000 | .000 | .000 | .000 |
| C2 | .920 | .718 | 1.141 | .000 |
| C1 | .806 | .629 | 1.000 | .000 |

## Standardized Total Effects (Group number 1 - Default model)

|  | A | B | C | D |
| --- | --- | --- | --- | --- |
| B | .686 | .000 | .000 | .000 |
| C | .648 | .474 | .000 | .000 |
| D | .386 | .282 | .595 | .000 |
| A4 | .735 | .000 | .000 | .000 |
| D4 | .266 | .194 | .410 | .688 |
| D3 | .279 | .204 | .430 | .723 |
| D2 | .281 | .205 | .433 | .728 |
| D1 | .246 | .180 | .380 | .638 |
| B1 | .488 | .712 | .000 | .000 |
| B2 | .534 | .778 | .000 | .000 |
| B3 | .410 | .598 | .000 | .000 |
| A1 | .726 | .000 | .000 | .000 |
| A2 | .652 | .000 | .000 | .000 |
| A3 | .718 | .000 | .000 | .000 |
| C2 | .513 | .375 | .792 | .000 |
| C1 | .422 | .309 | .651 | .000 |

## Direct Effects (Group number 1 - Default model)

|  | A | B | C | D |
| --- | --- | --- | --- | --- |
| B | .642 | .000 | .000 | .000 |
| C | .402 | .629 | .000 | .000 |
| D | .000 | .000 | .561 | .000 |
| A4 | 1.087 | .000 | .000 | .000 |
| D4 | .000 | .000 | .000 | 1.013 |
| D3 | .000 | .000 | .000 | 1.171 |
| D2 | .000 | .000 | .000 | 1.051 |
| D1 | .000 | .000 | .000 | 1.000 |
| B1 | .000 | 1.195 | .000 | .000 |
| B2 | .000 | 1.591 | .000 | .000 |
| B3 | .000 | 1.000 | .000 | .000 |
| A1 | 1.934 | .000 | .000 | .000 |
| A2 | .903 | .000 | .000 | .000 |
| A3 | 1.000 | .000 | .000 | .000 |
| C2 | .000 | .000 | 1.141 | .000 |
| C1 | .000 | .000 | 1.000 | .000 |

## Standardized Direct Effects (Group number 1 - Default model)

|  | A | B | C | D |
| --- | --- | --- | --- | --- |
| B | .686 | .000 | .000 | .000 |
| C | .324 | .474 | .000 | .000 |
| D | .000 | .000 | .595 | .000 |
| A4 | .735 | .000 | .000 | .000 |
| D4 | .000 | .000 | .000 | .688 |
| D3 | .000 | .000 | .000 | .723 |
| D2 | .000 | .000 | .000 | .728 |
| D1 | .000 | .000 | .000 | .638 |
| B1 | .000 | .712 | .000 | .000 |
| B2 | .000 | .778 | .000 | .000 |
| B3 | .000 | .598 | .000 | .000 |
| A1 | .726 | .000 | .000 | .000 |
| A2 | .652 | .000 | .000 | .000 |
| A3 | .718 | .000 | .000 | .000 |
| C2 | .000 | .000 | .792 | .000 |
| C1 | .000 | .000 | .651 | .000 |

## Indirect Effects (Group number 1 - Default model)

|  | A | B | C | D |
| --- | --- | --- | --- | --- |
| B | .000 | .000 | .000 | .000 |
| C | .404 | .000 | .000 | .000 |
| D | .452 | .353 | .000 | .000 |
| A4 | .000 | .000 | .000 | .000 |
| D4 | .458 | .357 | .568 | .000 |
| D3 | .529 | .413 | .656 | .000 |
| D2 | .475 | .371 | .589 | .000 |
| D1 | .452 | .353 | .561 | .000 |
| B1 | .767 | .000 | .000 | .000 |
| B2 | 1.022 | .000 | .000 | .000 |
| B3 | .642 | .000 | .000 | .000 |
| A1 | .000 | .000 | .000 | .000 |
| A2 | .000 | .000 | .000 | .000 |
| A3 | .000 | .000 | .000 | .000 |
| C2 | .920 | .718 | .000 | .000 |
| C1 | .806 | .629 | .000 | .000 |

## Standardized Indirect Effects (Group number 1 - Default model)

|  | A | B | C | D |
| --- | --- | --- | --- | --- |
| B | .000 | .000 | .000 | .000 |
| C | .325 | .000 | .000 | .000 |
| D | .386 | .282 | .000 | .000 |
| A4 | .000 | .000 | .000 | .000 |
| D4 | .266 | .194 | .410 | .000 |
| D3 | .279 | .204 | .430 | .000 |
| D2 | .281 | .205 | .433 | .000 |
| D1 | .246 | .180 | .380 | .000 |
| B1 | .488 | .000 | .000 | .000 |
| B2 | .534 | .000 | .000 | .000 |
| B3 | .410 | .000 | .000 | .000 |
| A1 | .000 | .000 | .000 | .000 |
| A2 | .000 | .000 | .000 | .000 |
| A3 | .000 | .000 | .000 | .000 |
| C2 | .513 | .375 | .000 | .000 |
| C1 | .422 | .309 | .000 | .000 |

## Modification Indices (Group number 1 - Default model)

## Covariances: (Group number 1 - Default model)

|  |  |  | M.I. | Par Change |
| --- | --- | --- | --- | --- |
| e10 | <--> | A | 7.175 | .022 |
| e10 | <--> | e15 | 7.858 | -.025 |
| e5 | <--> | e10 | 9.410 | .029 |
| e6 | <--> | e15 | 4.830 | .018 |
| e6 | <--> | e16 | 4.628 | .018 |
| e6 | <--> | e13 | 4.435 | .021 |
| e7 | <--> | e16 | 6.821 | -.020 |
| e7 | <--> | e10 | 4.035 | -.020 |
| e7 | <--> | e5 | 4.288 | .017 |
| e1 | <--> | e4 | 4.060 | .022 |
| e1 | <--> | e13 | 6.900 | -.037 |
| e1 | <--> | e10 | 7.708 | .043 |
| e1 | <--> | e5 | 5.118 | .028 |
| e3 | <--> | e2 | 7.005 | .016 |

## Variances: (Group number 1 - Default model)

|  |  |  | M.I. | Par Change |
| --- | --- | --- | --- | --- |

## Regression Weights: (Group number 1 - Default model)

|  |  |  | M.I. | Par Change |
| --- | --- | --- | --- | --- |
| D1 | <--- | A | 7.175 | .209 |
| D1 | <--- | B | 4.753 | .185 |
| D1 | <--- | A4 | 4.179 | .098 |
| D1 | <--- | B1 | 10.996 | .150 |
| D1 | <--- | A1 | 12.186 | .093 |
| B1 | <--- | D1 | 4.323 | .066 |
| B2 | <--- | D | 5.307 | .147 |
| B2 | <--- | D4 | 7.915 | .110 |
| B2 | <--- | D2 | 4.053 | .080 |
| B3 | <--- | D | 6.107 | -.146 |
| B3 | <--- | D4 | 6.927 | -.095 |
| B3 | <--- | D1 | 8.163 | -.097 |
| C1 | <--- | A1 | 4.384 | -.058 |

## Minimization History (Default model)

| Iteration |  | Negative eigenvalues | Condition # | Smallest eigenvalue | Diameter | F | NTries | Ratio |
| --- | --- | --- | --- | --- | --- | --- | --- | --- |
| 0 | e | 8 |  | -.362 | 9999.000 | 2148.923 | 0 | 9999.000 |
| 1 | e | 3 |  | -.039 | 2.403 | 786.256 | 20 | .531 |
| 2 | e | 0 | 66.356 |  | 1.463 | 284.540 | 5 | .708 |
| 3 | e | 0 | 50.171 |  | .556 | 204.465 | 3 | .000 |
| 4 | e | 0 | 38.632 |  | .584 | 120.990 | 1 | 1.077 |
| 5 | e | 0 | 43.123 |  | .260 | 108.481 | 1 | 1.095 |
| 6 | e | 0 | 58.965 |  | .094 | 107.900 | 1 | 1.053 |
| 7 | e | 0 | 58.228 |  | .011 | 107.893 | 1 | 1.009 |
| 8 | e | 0 | 60.160 |  | .000 | 107.893 | 1 | 1.000 |

## Pairwise Parameter Comparisons (Default model)

## Variance-covariance Matrix of Estimates (Default model)

|  | par\_1 | par\_2 | par\_3 | par\_4 | par\_5 | par\_6 | par\_7 | par\_8 | par\_9 | par\_10 | par\_11 | par\_12 | par\_13 | par\_14 | par\_15 | par\_16 | par\_17 | par\_18 | par\_19 | par\_20 | par\_21 | par\_22 | par\_23 | par\_24 | par\_25 | par\_26 | par\_27 | par\_28 | par\_29 | par\_30 |
| --- | --- | --- | --- | --- | --- | --- | --- | --- | --- | --- | --- | --- | --- | --- | --- | --- | --- | --- | --- | --- | --- | --- | --- | --- | --- | --- | --- | --- | --- | --- |
| par\_1 | .010 |
| par\_2 | .000 | .005 |
| par\_3 | .000 | .005 | .022 |
| par\_4 | .000 | .000 | .000 | .021 |
| par\_5 | .000 | .000 | .000 | .010 | .012 |
| par\_6 | .000 | .000 | .000 | .000 | .000 | .008 |
| par\_7 | .000 | .000 | .000 | .000 | .000 | .005 | .010 |
| par\_8 | .000 | .000 | .000 | .000 | .000 | .004 | .005 | .008 |
| par\_9 | .000 | .003 | .007 | .000 | .000 | .000 | .000 | .000 | .007 |
| par\_10 | -.001 | .001 | .002 | -.001 | .000 | .000 | .000 | .000 | .001 | .011 |
| par\_11 | .000 | .002 | .004 | -.006 | -.004 | .000 | .000 | .000 | .002 | .001 | .005 |
| par\_12 | -.004 | .000 | .000 | .007 | .004 | .000 | .000 | .000 | .000 | -.009 | -.002 | .017 |
| par\_13 | .002 | .000 | .000 | .000 | .000 | -.003 | -.003 | -.003 | .000 | -.001 | .000 | -.001 | .005 |
| par\_14 | .000 | -.001 | -.001 | .000 | .000 | .000 | .000 | .000 | -.001 | .000 | .000 | .000 | .000 | .000 |
| par\_15 | .000 | .000 | .000 | -.001 | -.001 | .000 | .000 | .000 | .000 | .000 | .000 | .000 | .000 | .000 | .000 |
| par\_16 | -.001 | .000 | .000 | .000 | .000 | .000 | .000 | .000 | .000 | .000 | .000 | .000 | .000 | .000 | .000 | .000 |
| par\_17 | .000 | .000 | .000 | .000 | .000 | -.001 | -.001 | -.001 | .000 | .000 | .000 | .000 | .000 | .000 | .000 | .000 | .000 |
| par\_18 | .001 | .000 | .000 | .000 | .000 | .000 | .000 | .000 | .000 | .000 | .000 | .000 | .000 | .000 | .000 | .000 | .000 | .000 |
| par\_19 | -.001 | .000 | .000 | .000 | .000 | .000 | .000 | .000 | .000 | .000 | .000 | .000 | .000 | .000 | .000 | .000 | .000 | .000 | .000 |
| par\_20 | .000 | .000 | .000 | .000 | .000 | .000 | .000 | .000 | .000 | .000 | .000 | .000 | .000 | .000 | .000 | .000 | .000 | .000 | .000 | .000 |
| par\_21 | .000 | .000 | .000 | .000 | .000 | .000 | .000 | .000 | .000 | .000 | .000 | .000 | .000 | .000 | .000 | .000 | .000 | .000 | .000 | .000 | .000 |
| par\_22 | .000 | .000 | -.001 | .000 | .000 | .000 | .000 | .000 | .000 | .000 | .000 | .000 | .000 | .000 | .000 | .000 | .000 | .000 | .000 | .000 | .000 | .001 |
| par\_23 | .000 | .000 | .000 | .000 | .000 | .000 | .000 | .000 | .000 | .000 | .000 | .000 | .000 | .000 | .000 | .000 | .000 | .000 | .000 | .000 | .000 | .000 | .000 |
| par\_24 | .000 | .000 | .000 | -.001 | .000 | .000 | .000 | .000 | .000 | .000 | .000 | .000 | .000 | .000 | .000 | .000 | .000 | .000 | .000 | .000 | .000 | .000 | .000 | .000 |
| par\_25 | .000 | .000 | .000 | .000 | .000 | .000 | .000 | .000 | .000 | .000 | .000 | .000 | .000 | .000 | .000 | .000 | .000 | .000 | .000 | .000 | .000 | .000 | .000 | .000 | .000 |
| par\_26 | .000 | .000 | .000 | .000 | .000 | .000 | .000 | .000 | .000 | .000 | .000 | .000 | .000 | .000 | .000 | .000 | .000 | .000 | .000 | .000 | .000 | .000 | .000 | .000 | .000 | .000 |
| par\_27 | .000 | .000 | .000 | .000 | .000 | .000 | .000 | .000 | .000 | .000 | .000 | .000 | .000 | .000 | .000 | .000 | .000 | .000 | .000 | .000 | .000 | .000 | .000 | .000 | .000 | .000 | .000 |
| par\_28 | .000 | .000 | .000 | .000 | .000 | .000 | .000 | .000 | .000 | .000 | .000 | .000 | .000 | .000 | .000 | .000 | .000 | .000 | .000 | .000 | .000 | .000 | .000 | .000 | .000 | .000 | .000 | .000 |
| par\_29 | .000 | .000 | .000 | .000 | .000 | .000 | .000 | .000 | .000 | .000 | .000 | .000 | .000 | .000 | .000 | .000 | .000 | .000 | .000 | .000 | .000 | .000 | .000 | .000 | .000 | .000 | .000 | .000 | .000 |
| par\_30 | .000 | .000 | .000 | .000 | .000 | .000 | .000 | .000 | .000 | .000 | .000 | .000 | .000 | .000 | .000 | .000 | .000 | .000 | .000 | .000 | .000 | .000 | .000 | .000 | .000 | .000 | .000 | .000 | .000 | .000 |

## Critical Ratios for Differences between Parameters (Default model)

|  | par\_1 | par\_2 | par\_3 | par\_4 | par\_5 | par\_6 | par\_7 | par\_8 | par\_9 | par\_10 | par\_11 | par\_12 | par\_13 | par\_14 | par\_15 | par\_16 | par\_17 | par\_18 | par\_19 | par\_20 | par\_21 | par\_22 | par\_23 | par\_24 | par\_25 | par\_26 | par\_27 | par\_28 | par\_29 | par\_30 |
| --- | --- | --- | --- | --- | --- | --- | --- | --- | --- | --- | --- | --- | --- | --- | --- | --- | --- | --- | --- | --- | --- | --- | --- | --- | --- | --- | --- | --- | --- | --- |
| par\_1 | .000 |
| par\_2 | -1.937 | .000 |
| par\_3 | 4.493 | 7.768 | .000 |
| par\_4 | 2.571 | 4.263 | -1.655 | .000 |
| par\_5 | .374 | 2.254 | -4.067 | -3.445 | .000 |
| par\_6 | -.672 | 1.293 | -5.134 | -3.183 | -1.026 | .000 |
| par\_7 | .212 | 2.151 | -4.277 | -2.383 | -.165 | 1.378 | .000 |
| par\_8 | -.953 | .959 | -5.347 | -3.405 | -1.296 | -.450 | -1.810 | .000 |
| par\_9 | -.420 | 2.258 | -6.908 | -3.042 | -.802 | .294 | -.646 | .608 | .000 |
| par\_10 | -4.827 | -4.200 | -9.160 | -6.521 | -5.255 | -4.689 | -5.243 | -4.405 | -5.576 | .000 |
| par\_11 | -4.084 | -3.099 | -9.359 | -4.898 | -3.534 | -3.578 | -4.261 | -3.236 | -5.099 | 1.964 | .000 |
| par\_12 | -2.711 | -1.835 | -6.614 | -6.167 | -3.914 | -2.668 | -3.272 | -2.429 | -2.982 | 1.062 | -.077 | .000 |
| par\_13 | -5.861 | -3.410 | -8.447 | -6.475 | -4.976 | -3.658 | -4.166 | -3.371 | -4.932 | 1.199 | -.808 | -.438 | .000 |
| par\_14 | -10.382 | -10.000 | -11.768 | -10.270 | -10.062 | -10.482 | -10.412 | -10.020 | -10.948 | -2.727 | -6.853 | -4.001 | -6.391 | .000 |
| par\_15 | -11.003 | -11.768 | -12.809 | -10.262 | -10.114 | -11.178 | -11.021 | -10.709 | -12.783 | -3.369 | -8.545 | -4.335 | -7.249 | -3.614 | .000 |
| par\_16 | -9.923 | -11.309 | -12.607 | -10.431 | -10.325 | -10.836 | -10.736 | -10.375 | -12.345 | -3.127 | -7.781 | -4.267 | -6.332 | -1.607 | 1.634 | .000 |
| par\_17 | -10.582 | -11.020 | -12.475 | -10.323 | -10.147 | -9.626 | -9.668 | -9.247 | -12.083 | -2.901 | -7.480 | -4.070 | -6.902 | -.609 | 2.577 | .895 | .000 |
| par\_18 | -9.769 | -9.141 | -11.569 | -9.419 | -8.930 | -9.081 | -9.176 | -8.620 | -10.413 | -1.680 | -5.644 | -3.028 | -4.871 | 5.046 | 8.247 | 5.501 | 5.398 | .000 |
| par\_19 | -9.453 | -10.527 | -12.226 | -10.117 | -9.820 | -10.150 | -10.131 | -9.706 | -11.663 | -2.573 | -6.981 | -3.914 | -6.135 | .986 | 4.126 | 2.364 | 1.405 | -3.504 | .000 |
| par\_20 | -10.495 | -11.335 | -12.690 | -10.324 | -10.160 | -10.617 | -10.525 | -10.150 | -12.522 | -2.884 | -7.669 | -4.048 | -6.544 | -.369 | 4.015 | 1.482 | .329 | -5.891 | -1.465 | .000 |
| par\_21 | -10.324 | -10.612 | -12.420 | -10.210 | -9.999 | -10.419 | -10.350 | -9.952 | -12.035 | -2.703 | -7.274 | -3.920 | -6.299 | .656 | 5.249 | 2.479 | 1.297 | -5.021 | -.535 | 1.391 | .000 |
| par\_22 | -7.549 | -7.003 | -9.953 | -8.391 | -7.483 | -7.361 | -7.682 | -6.936 | -8.243 | -.420 | -3.599 | -2.034 | -2.671 | 7.649 | 9.481 | 8.245 | 7.591 | 3.661 | 6.553 | 7.707 | 7.175 | .000 |
| par\_23 | -9.770 | -10.068 | -11.988 | -10.081 | -9.717 | -9.806 | -9.814 | -9.347 | -11.231 | -2.199 | -6.343 | -3.586 | -5.550 | 3.307 | 6.776 | 4.744 | 3.677 | -2.418 | 1.916 | 4.313 | 3.153 | -5.620 | .000 |
| par\_24 | -9.881 | -10.163 | -12.076 | -9.519 | -9.624 | -9.890 | -9.902 | -9.430 | -11.328 | -2.331 | -6.848 | -3.596 | -5.649 | 2.161 | 5.450 | 3.561 | 2.609 | -2.730 | 1.077 | 2.798 | 1.860 | -5.688 | -.619 | .000 |
| par\_25 | -9.276 | -9.365 | -11.658 | -9.507 | -9.039 | -9.671 | -9.742 | -9.184 | -10.610 | -1.787 | -5.839 | -3.178 | -4.696 | 4.924 | 8.496 | 6.311 | 4.703 | -.431 | 3.539 | 5.897 | 4.915 | -4.096 | 2.083 | 2.434 | .000 |
| par\_26 | -9.997 | -10.398 | -12.153 | -10.007 | -9.718 | -9.707 | -10.082 | -9.687 | -11.533 | -2.442 | -6.835 | -3.713 | -5.891 | 1.980 | 5.876 | 3.532 | 2.488 | -3.427 | .764 | 2.749 | 1.625 | -6.314 | -1.296 | -.459 | -3.130 | .000 |
| par\_27 | -9.573 | -9.771 | -11.860 | -9.721 | -9.322 | -9.561 | -9.210 | -9.045 | -10.975 | -2.066 | -6.239 | -3.407 | -5.392 | 3.588 | 7.040 | 4.899 | 3.986 | -1.614 | 2.360 | 4.405 | 3.435 | -4.950 | .711 | 1.214 | -1.192 | 1.747 | .000 |
| par\_28 | -9.768 | -10.054 | -11.991 | -9.838 | -9.493 | -9.866 | -9.786 | -9.005 | -11.229 | -2.221 | -6.503 | -3.531 | -5.574 | 3.101 | 6.964 | 4.562 | 3.523 | -2.379 | 1.799 | 4.006 | 2.914 | -5.554 | -.042 | .597 | -2.008 | 1.112 | -.714 | .000 |
| par\_29 | -10.430 | -11.006 | -12.381 | -10.285 | -10.103 | -10.532 | -10.451 | -10.067 | -11.691 | -2.800 | -7.393 | -3.994 | -6.436 | .018 | 4.305 | 1.844 | .704 | -5.487 | -1.073 | .504 | -.753 | -7.709 | -3.795 | -2.384 | -5.431 | -2.256 | -3.963 | -3.504 | .000 |
| par\_30 | -10.141 | -10.612 | -12.243 | -10.230 | -9.643 | -10.246 | -10.201 | -9.784 | -11.732 | -2.548 | -7.033 | -3.865 | -6.081 | 1.306 | 5.201 | 2.879 | 1.836 | -4.131 | .115 | 2.003 | .824 | -6.858 | -2.196 | -1.018 | -3.975 | -.771 | -2.580 | -1.985 | 1.494 | .000 |

## Model Fit Summary

## CMIN

| Model | NPAR | CMIN | DF | P | CMIN/DF |
| --- | --- | --- | --- | --- | --- |
| Default model | 30 | 107.893 | 61 | .000 | 1.769 |
| Saturated model | 91 | .000 | 0 |
| Independence model | 13 | 2028.542 | 78 | .000 | 26.007 |

## RMR, GFI

| Model | RMR | GFI | AGFI | PGFI |
| --- | --- | --- | --- | --- |
| Default model | .014 | .965 | .948 | .647 |
| Saturated model | .000 | 1.000 |  |  |
| Independence model | .105 | .423 | .327 | .363 |

## Baseline Comparisons

| Model | NFI Delta1 | RFI rho1 | IFI Delta2 | TLI rho2 | CFI |
| --- | --- | --- | --- | --- | --- |
| Default model | .947 | .932 | .976 | .969 | .976 |
| Saturated model | 1.000 |  | 1.000 |  | 1.000 |
| Independence model | .000 | .000 | .000 | .000 | .000 |

## Parsimony-Adjusted Measures

| Model | PRATIO | PNFI | PCFI |
| --- | --- | --- | --- |
| Default model | .782 | .740 | .763 |
| Saturated model | .000 | .000 | .000 |
| Independence model | 1.000 | .000 | .000 |

## NCP

| Model | NCP | LO 90 | HI 90 |
| --- | --- | --- | --- |
| Default model | 46.893 | 21.805 | 79.832 |
| Saturated model | .000 | .000 | .000 |
| Independence model | 1950.542 | 1807.436 | 2101.007 |

## FMIN

| Model | FMIN | F0 | LO 90 | HI 90 |
| --- | --- | --- | --- | --- |
| Default model | .237 | .103 | .048 | .175 |
| Saturated model | .000 | .000 | .000 | .000 |
| Independence model | 4.449 | 4.278 | 3.964 | 4.607 |

## RMSEA

| Model | RMSEA | LO 90 | HI 90 | PCLOSE |
| --- | --- | --- | --- | --- |
| Default model | .041 | .028 | .054 | .875 |
| Independence model | .234 | .225 | .243 | .000 |

## AIC

| Model | AIC | BCC | BIC | CAIC |
| --- | --- | --- | --- | --- |
| Default model | 167.893 | 169.793 | 291.634 | 321.634 |
| Saturated model | 182.000 | 187.765 | 557.346 | 648.346 |
| Independence model | 2054.542 | 2055.365 | 2108.163 | 2121.163 |

## ECVI

| Model | ECVI | LO 90 | HI 90 | MECVI |
| --- | --- | --- | --- | --- |
| Default model | .368 | .313 | .440 | .372 |
| Saturated model | .399 | .399 | .399 | .412 |
| Independence model | 4.506 | 4.192 | 4.836 | 4.507 |

## HOELTER

| Model | HOELTER .05 | HOELTER .01 |
| --- | --- | --- |
| Default model | 340 | 379 |
| Independence model | 23 | 25 |

## Execution time summary

|  |  |
| --- | --- |
| Minimization: | .021 |
| Miscellaneous: | .229 |
| Bootstrap: | .000 |
| Total: | .250 |
