# Supplementary material for: Self-health promotion: A study on the mode of acquiring sports health knowledge and skills among older adults members of sports communities
Source: PLoS One. 2024 Jul 11;19(7):e0304814. doi: 10.1371/journal.pone.0304814 (PMC11239043; doi:10.1371/journal.pone.0304814)
Supplement: S1 File — (ZIP) [file pone.0304814.s001.zip › data-Self-health Promotion/questionnaire.docx]

**Survey questionnaire**

**Self-Health promotion: a study on the mode of acquiring sports health knowledge and skills among older adults members of sports communities**

The sports community in the questionnaire mainly refers to older adults fitness sports community, with stable participants(≥3 people), regular community fitness activities, and community activity plans.

Researchers explain the purpose and content of the study and fill in the rules.

**一、Personal data of older adults(.Please call according to your situation√.)**

1.Inclusion criteria:

(1) Older adults residing in Luoyang city for at least 1 year ; (2) Age: ≥60 years; (3) Well-informed about and consenting to the research topic, adhering to the principle of voluntary participation; (4) Proficient in communication, free of mental disorders, and possessing a reasonable level of physical activity ability; (5) older adults respondents must have at least 6 months' experience in community sports activities.

Number of years devoted to sports: The participants under investigation possess a minimum of six months of experience in community activities. Based on the fundamental conditions of the sports community, the 'Number of years devoted to sports' is determined using a range-defined approach, facilitating statistical analysis. The scholars involved in the assessment posit that the method for defining 'Number of years devoted to sports' is as follows: 0.6 ≤ 'Number of years devoted to sports' < 1 year, defined as 0.6 years; 1 ≤ 'Number of years devoted to sports' < 2 years, defined as 1 year; 2 ≤ 'Number of years devoted to sports' < 3 years, defined as 2 years; 3 ≤ 'Number of years devoted to sports', defined as ≥3 years.

2. Gender □female □male.

3. Age: □60~62 □63~64 □65~66 □≥67.

4.Educational background: □Primary school education or below □Middle (or high) school education □Junior college education or above.

5.Health status : □unhealthy status(feeling unwell or ill in the last eight weeks); □ healthy status.

6. Number of years devoted to sports: □0.6 □1 □2 □≥3.

**二、 questionnaire**

The purpose of this questionnaire is to collect relevant data from participants in t older adults sports community and to construct a theoretical model of their acquisition of physical health knowledge and skills. Please answer the questions according to their meaning. The questionnaire uses a Likert 5-point scale to indicate the self-perceived level of agreement. 1-5: Strongly agree, agree, no opinion, disagree, strongly disagree. Please rate your response according to your own situation. Please call according to your situation√.

|  | ***Sports Community Culture Scale*** | | | | | |
| --- | --- | --- | --- | --- | --- | --- |
| **No** | **content** | **1** | **2** | **3** | **4** | **5** |
| 1 | Joining a sports community requires members to possess a certain level of knowledge and skills in physical fitness. |  |  |  |  |  |
| 2 | Before or during the social movement process, the participating members often brainstorm and brainstorm discussions of sports health knowledge and skills in the campaign and the collective effort to explore solutions or make things perfect. |  |  |  |  |  |
| 3 | The sports community I belong to has norms that organize members to share knowledge and skills related to physical health, as well as norms that care for the health of others. |  |  |  |  |  |
| 4 | In interactions with other community members, one can subtly perceive others' knowledge and experience in sports and health and continue to learn. |  |  |  |  |  |
| 5 | The sports community I participate in has experienced members with extensive knowledge and skills in sports and health, and they are willing to share their experiences selflessly. |  |  |  |  |  |
| 6 | I participate in sports community groups and online courses (or other media groups) and can acquire the sports and health knowledge and skills I need. |  |  |  |  |  |
| 7 | Sports community members often discuss and share knowledge and skills related to sports and health outside of community activities. |  |  |  |  |  |
| 8 | I can quickly obtain the necessary sports and health knowledge and skills from the sports community I participate in |  |  |  |  |  |
| 9 | I like the sports community and am willing to pay for it. |  |  |  |  |  |
| 10 | If I am invited by teammates in the community, I am always happy to participate in the fitness and wellness programs they plan. |  |  |  |  |  |
| 11 | During the social movement process, I like the feeling of mutual care and sharing with teammates. |  |  |  |  |  |
| 12 | To be accepted and recognized as a member of my community, I am willing to diligently study relevant sports and health knowledge and skills. |  |  |  |  |  |
| 13 | To meet the requirements of the sports community, I am willing to diligently study relevant sports health knowledge and skills. |  |  |  |  |  |
| 14 | The development of the sports community is important to me. |  |  |  |  |  |
| 15 | I am willing to contribute to maintaining harmony in the sports community. |  |  |  |  |  |
| 16 | I will enrich community culture and ensure that participants experience the value of life. |  |  |  |  |  |
| ***Health Knowledge and Skill Pursuit Scale*** | | | | | | |
| 1 | To gain more confidence in sports, I constantly acquire knowledge and skills related to physical fitness. |  |  |  |  |  |
| 2 | In the community movement, I need to further improve my physical health knowledge and skills to face various challenges and difficulties encountered in sports. |  |  |  |  |  |
| 3 | I must further improve my health promotion plan in response to social movements. |  |  |  |  |  |
| 4 | The history of social movement proves that my relevant sports and health knowledge and skills can meet various fitness challenges. |  |  |  |  |  |
| 5 | The social and cultural movement and the healthy environment of the community make me feel thrilled and fulfilled. |  |  |  |  |  |
| 6 | Due to the social movement of Yinyuan, I have rich knowledge and skills in sports and health, which has made my life feel very fulfilling. |  |  |  |  |  |
| 7 | Throughout the social movement, there have been new sports and health knowledge and skills experiences at any time, making my life more fulfilling. |  |  |  |  |  |
| 8 | My life is very fulfilling because I participate in social movements and share my knowledge and skills about sports and health with others. |  |  |  |  |  |
| 9 | Community sports have allowed me to showcase my knowledge and expertise in sports and health. |  |  |  |  |  |
| 10 | I can show my unique intellectual and comprehensive ability during the social movement. |  |  |  |  |  |
| 11 | Participating in community movements can show one's contribution to others. |  |  |  |  |  |
| 12 | The challenging nature of social movement allows me to show a brighter and healthier self. |  |  |  |  |  |
| ***Motivation to Enhance Sports Health Knowledge and Skills Scale*** | | | | | | |
| 1 | In the early stages of social movement, evaluating one's physical health knowledge and skills and planning for enrichment is essential. |  |  |  |  |  |
| 2 | Through participation in community sports, one will feel that their knowledge and skills in physical fitness are insufficient and need to be further enriched. |  |  |  |  |  |
| 3 | My peers or superiors will point out my deficiencies in sports and health knowledge and skills. |  |  |  |  |  |
| 4 | To show myself, I still need to continuously enrich my knowledge, skills, and sports experience in various ways. |  |  |  |  |  |
| 5 | I interact with like-minded people, and I can observe and realize that my sports health knowledge and skills still need improvement. |  |  |  |  |  |
| 6 | The more group sports experiences one has, the more sports health knowledge and skills one consciously wants to learn. |  |  |  |  |  |
| 7 | New knowledge and skills in sports and health can help promote the benefits of one's own sports and health. |  |  |  |  |  |
| 8 | Novel health and sports knowledge and skills can make me a person with a say. |  |  |  |  |  |
| 9 | The changes in the social and cultural environment, or the emergence of new health skills and knowledge, make my fitness and health content brimming with unforeseen joy and the happiness of professional growth, with a never-ending drive to learn. |  |  |  |  |  |
| 10 | Every social movement represents a new challenge and learning opportunity, and one should prepare relevant sports and health knowledge and skills accordingly. |  |  |  |  |  |
| 11 | I am still willing to learn and prepare for new knowledge and skills in sports and health that may not be applicable. |  |  |  |  |  |
| 12 | Conducting new and complex community health promotion activities represents a new challenge and learning opportunity. I will engage in the learning of relevant sports health knowledge and skills. |  |  |  |  |  |
| ***Behavior of Acquiring Sports Health Knowledge and Skills Scal*** | | | | | | |
| 1 | Due to the social media movement, I often browse media websites to absorb knowledge and skills in sports and health. |  |  |  |  |  |
| 2 | I continuously participate in health promotion activities, experiencing and absorbing sports and health knowledge and skills during the process. |  |  |  |  |  |
| 3 | Due to social movements, I often acquire knowledge and skills related to physical health and sports from the same place. |  |  |  |  |  |
| 4 | Due to the social movement, I often targeted to improve my sports and health knowledge, skills, and experiences. |  |  |  |  |  |
| 5 | Participating in community sports activities has allowed me to gain more knowledge and skills related to sports and health. |  |  |  |  |  |
| 6 | Participating in community movements has enhanced my understanding and awareness of the region and the community's human health and well-being environment. |  |  |  |  |  |
| 7 | Participating in community sports taught me how to collaborate with others to share knowledge and skills about physical health. |  |  |  |  |  |
| 8 | Through participating in community sports activities, I have gained a better understanding of the necessary physical and health knowledge and skills that should be prepared before participating in sports. |  |  |  |  |  |
| 9 | Due to the social movement, I am willing to put in more effort to make my knowledge and skills in physical fitness more professional. |  |  |  |  |  |
| 10 | Due to social movements, I often share knowledge and skills about sports and health with others, and I feel happy. |  |  |  |  |  |
| 11 | In the social movement context, when I am teaching or sharing knowledge and skills related to sports and health, I listen and learn with great attention. |  |  |  |  |  |
| 12 | I enjoy applying the new sports and health knowledge and skills learned in the social movement to sports. |  |  |  |  |  |
| 13 | Due to the social movement, I need to acquire knowledge and skills in sports and health. |  |  |  |  |  |
| 14 | The acquisition of sports and health knowledge and skills through the social movement of Yinyuan is very appealing to me. |  |  |  |  |  |
| 15 | The acquisition of sports and health knowledge and skills through the social movement of Yinyuan is valuable to me. |  |  |  |  |  |
| 16 | When it comes to sports and health knowledge and skills, I will put in effort. |  |  |  |  |  |
| 17 | Being familiar with sports and health knowledge and skills is purposeful in the context of social movement. |  |  |  |  |  |

Over！！
